# Phonetic Realizations of Metrical Structure in Tone Languages: Evidence From Chinese Dialects

**DOI:** 10.3389/fpsyg.2022.945973

**Published:** 2022-07-13

**Authors:** Chengyu Guo, Fei Chen

**Affiliations:** School of Foreign Languages, Hunan University, Changsha, China

**Keywords:** metrical structure, Chinese dialects, pitch, duration, metrical tone sandhi

## Abstract

In tone languages, some case studies showed that the word-level tonal representation was closely related to the underlying metrical pattern. Based on different tonal patterns in prosodic units, the metrical structures could generally be divided into the left- and right-dominant types in Chinese dialects. Yet the cross-dialectal phonetic realizations (e.g., duration and pitch) between or within these two metrical structures were still unrevealed. The current study investigated the duration and pitch realizations of disyllabic prosodic words in Changsha and Chengdu dialects (the left-dominant structure), and in Fuzhou and Xiamen dialects (the right-dominant structure). Results showed that not all the duration patterns across four Chinese dialects were sensitive to different metrical structures, indicating that the duration might not be the universal cue for metrical prominence in Chinese dialects. In terms of pitch realization across all the four Chinese dialects, level tones (sometimes falling tones) generally appeared in the metrically weak unit, while underlying pitch forms appeared in the metrically strong unit. Compared with duration, pitch might be more robust for prosodic realizations of metrical structures in Chinese dialects. Furthermore, there was an interaction between duration and pitch patterns in Chinese dialects, which could shed new light on the phenomenon of “metrical tone sandhi”. Meanwhile, this study also provides some references for the judgment of the metrical stress and prosodic realizations in other Chinese dialects.

## Introduction

According to the function of prosodic elements at the word level, it is proposed that the world languages can be divided into different types such as tone languages and stress accent languages (Hyman, 2006, 2009). In tone languages like Chinese, the issues concerning tonal inventory and word stress, especially in Mandarin, have been extensively discussed in previous studies (Chao, 1968; Cheng, 1973; Duanmu, 2007; Zhang, 2016; Feng, 2017). However, the metrical structure (Liberman and Prince, 1977) and its phonetic realizations in different Chinese dialects were not fully understood. The current study presents phonetic realizations (i.e., duration and pitch) of left- and right-dominant metrical structures in four Chinese dialects with a cross-dialectal perspective.

It is not uncommon that the metrical structure is closely related to the tonal manifestation at the word level. To be more specific, the high tone (H tone) is more often linked to the metrically strong unit in tone languages such as Ayutla Mixtec (de Lacy, 2002), Kera (Pearce, 2006), and Moro (Jenks and Rose, 2011), as well as in pitch-accent languages such as Nguni (Downing, 1990) and Serbo-Croatian (Inkelas and Zec, 1988). Likewise, in Chinese dialects, some case studies consistently reported that the surface representation of lexical tones might be sensitive to prosodic prominence. Specifically, the surface tone in stressed syllables could be fully realized as its underlying pitch form (Lee, 1997; Kochanski et al., 2003; Sui, 2016), while in the prosodic weak unit undergoes pitch lowering or leveling in Chinese dialects such as Changsha dialect (Zhong, 2003), Chongming dialect (Chen, 2000), Fuzhou dialect (Wright, 1983), and Suzhou dialect (Zhu, 2021). More importantly, this pitch change might result in the reduction of the underlying tonal features, i.e., register and contour (Yip, 2002), and even trigger tonal merger in metrically weak mora or syllable. This tonal alternation could be named “metrical tone sandhi” (Zeng and Niu, 2006). Accordingly, the metrical structure of Chinese dialects could be categorized into the left- and right-dominant types based on the different pitch forms (citation/sandhi form) in metrically weak and strong positions (Yue-Hashimoto, 1987; Zhang, 2007).

Overall, the consensus reached in previous studies showed that the surface pitch realization was the key correlate of metrical structure in Chinese dialects. Besides, the duration might also act as a phonetic parameter indicating the prosodic strength in Mandarin (Chen and Xu, 2006; Xu, 2009). Although there was a dispute about the existence of prosodic contrast in Mandarin disyllabic words (Hoa, 1983; Duanmu, 1993; Xu and Wang, 2009; Zhang, 2016, 2021; Feng, 2017), the typical left-dominant (strong-weak) pattern was found in neutral-toned words with a long-short duration pattern (see 1a). The acoustic cue of the neutral-toned syllable in Mandarin is comparable to that of the unstressed syllable in English (Chen and Xu, 2006; Xu, 2009). In contrast, under the right-dominant structure, the duration might exhibit a short-long pattern (see 1b), since this duration pattern has been mentioned in the right-dominant dialects with impressionistic descriptions (Wright, 1983; Chan, 1985). Additionally, a short-long duration pattern is the canonical type for the iambic foot, according to the Iambic/Trochaic Law (Hayes, 1995). The question then arises whether or not the duration pattern in disyllabic words is also sensitive to the metrical structures across other Chinese dialects. To answer this question, more comprehensive research is needed to validate whether different duration patterns in Chinese dialects correspond with different metrical structures.


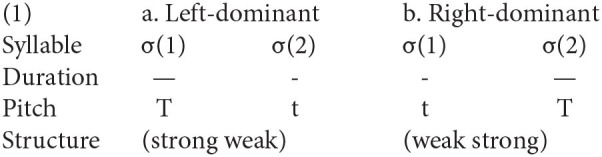


*Note*. “—” stands for a relatively longer duration, and “-” stands for a relatively shorter duration; “T” stands for the underlying pitch form, and “t” stands for the sandhi form.

Theoretically, the phonetic realization we speculated in (1) is seemingly symmetrical between two types of metrical structures. In terms of pitch realization, the underlying pitch form and sandhi form generally appear at σ(1) and σ(2), respectively, under the left-dominant structure, while occurring at σ(2) and σ(1) under the right-dominant structure. Likewise, in terms of duration realization, a long-short pattern and a short-long pattern symmetrically occur in the left- and right-dominant structures, respectively, according to our prediction (1). However, the actual phonetic realizations across Chinese dialects might be complex and diverse. It was reported that the underlying tone in the initial metrically strong syllable might spread rightwards to the weak syllable in the left-dominant structure [e.g., /kα sæ/ (artificial mountain) [51 33] → [53 31] in Tangsic dialect; Kennedy, 1953]. In this case, the surface tonal representation of two syllables in the left-dominant structure also could be the sandhi form. In other words, the pitch realizations between two metrical structures could be asymmetrical in Chinese dialects (Duanmu, 1995; Zhang, 2007). Furthermore, some cross-linguistic research has detected the durational asymmetry (Hayes, 1995; Gordon et al., 2018), that is, equally matched duration pattern (i.e., long-long or short-short pattern) under the trochaic foot (left-dominant structure), whereas the short-long duration pattern under the iambic foot (right-dominant structure). Overall, (1a) and (1b) are the ideally symmetrical realizations between two metrical types based on our assumption. Nevertheless, given various phonetic realizations for the left-dominant structure as reported in the literature, we wonder whether there are other diverse phonetic realizations for the right-dominant structure. Thus, to reveal the diversity of phonetic realizations for two types of metrical structures in Chinese dialects, a cross-dialectal investigation was conducted in the current study.

As mentioned above, different surface tonal representations between metrically weak and strong units have been seen as the key indicator of the metrical structure in Chinese dialects. The term “metrical tone sandhi” (Zeng and Niu, 2006) seems to be suitable to depict the phonological phenomenon that tone sandhi usually occurs in the prosodic weak unit. Still, some related issues remained understudied. For instance, it is unknown how the underlying tone interacts with different prosodic units specifically. Besides, the analyses of metrical tone sandhi in Chinse dialects were generally based on perceptual judgment and phonological description (Yue-Hashimoto, 1987; Zeng and Niu, 2006; Zhang, 2007). To tackle these problems, more empirical research and phonetic analyses should be carried out. Recently, a fine-grained method [Growth Curve Analysis (hereafter “GCA”); Mirman, 2014] of analyzing pitch contour has been introduced (Shi et al., 2020). The GCA could be used to compare the fine-grained differences over time in terms of pitch height, pitch slope, and pitch curvature. Therefore, GCA offers us a valuable chance to validate pitch realizations of metrical tone sandhi at the phonetic level.

The current study aimed at illustrating diverse phonetic realizations (i.e., duration and pitch) of disyllabic words under two metrical structures in Chinese by cross-dialectal comparisons. Two dialects under each left- and right-dominant structure (four dialects in total) were chosen since previous studies have proposed their metrical structures according to the tonal representation. Specifically, the representatives of the left-dominant structure were Changsha dialect (Zhong, 2003; Lin, 2011) and Chengdu dialect (Lin, 2006; Qin, 2012). In these two dialects, the underlying tone usually undergoes tone sandhi in the final position of disyllabic words. It should be noted that Changsha dialect also seems to show the right-dominant structure, with the tonal process occurring at the first syllable. However, this pattern is only limited to a few grammatical categories and beyond the scope of our study. Moreover, the chosen dialects under the right-dominant structure were Fuzhou dialect (Wright, 1983; Chan, 1985) and Xiamen dialect (Yue-Hashimoto, 1986; Hsieh, 2005), in which tone sandhi occurs at the initial position of disyllabic words.

**Figure 1:**
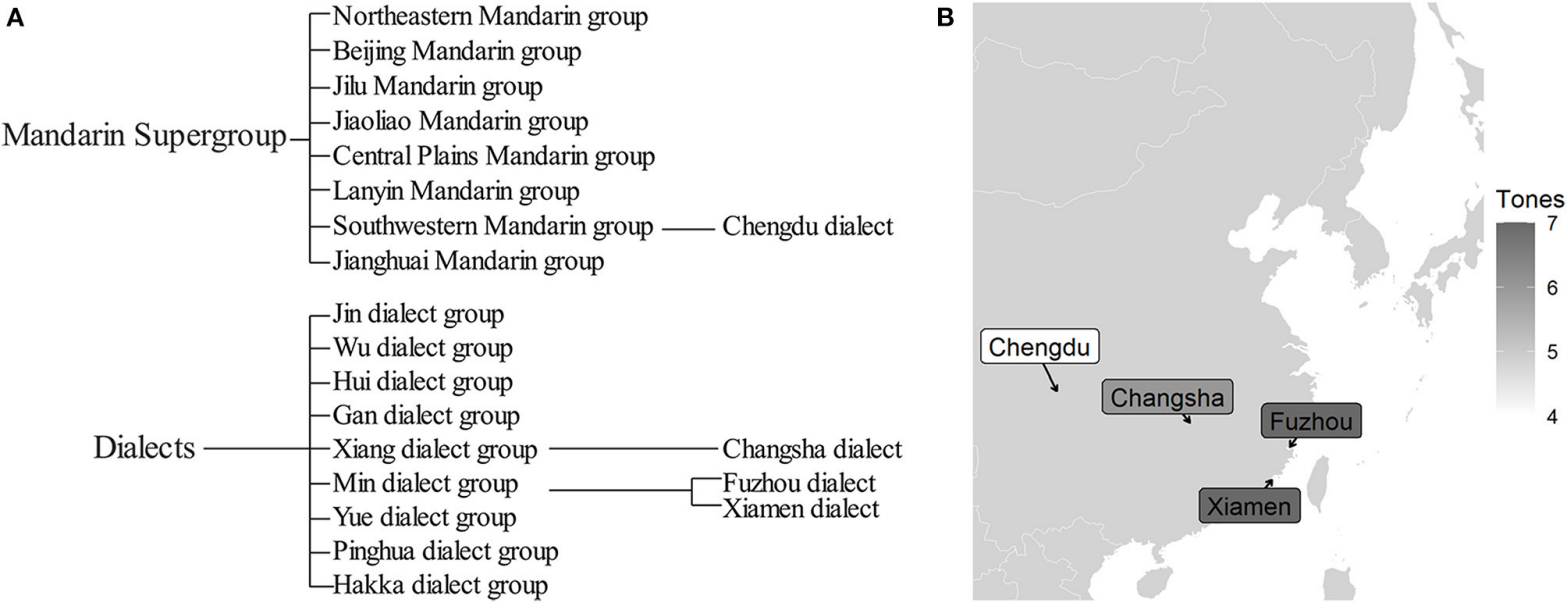
The classification **(A)** and geographic distribution **(B)** of four Chinese dialects in this study.

It should be noted that the genetic classification is different among the four Chinese dialects. According to Li et al. (1987) and Kurpaska (2010), Chengdu dialect belongs to the Southwestern Mandarin group, while the other three are classified into the southern Chinese dialects. To be more specific, Changsha dialect belongs to the Xiang dialect group; Fuzhou and Xiamen dialects belong to the Min dialect group. The classification and geographic distribution of the four dialects are shown in Figure 1.

Furthermore, the tone inventories of the four dialects are quite different (see Table 1). Chengdu dialect has four lexical tones (Qin, 2012); Changsha dialect has six (Zhong, 2003); both Fuzhou (Donohue, 2013) and Xiamen dialects (Chen, 1987) have seven. According to previous studies of phonological description (Chen, 1987; Zhong, 2003; Qin, 2012; Donohue, 2013), the relative tone values of the four dialects can be seen in Table 1. In Changsha and Chengdu dialects, their tonal inventories only include contour tones (i.e., rising or falling tones). Tone 6 and Tone 7 in Fuzhou and Xiamen dialects are checked tones, which are shorter than other lexical tones perceptually. In addition to contour tones, there is only one level tone in Fuzhou dialect, but two in Xiamen dialect. Apart from the checked tones, the syllable structures of Changsha, Fuzhou, and Xiamen dialects are similar, and tone-bearing units in these dialects are generally biomoraic rimes (Duanmu, 1990). The digits in Table 1 refer to lexical tone transcriptions in Chao's five-scale tone letters (Chao, 1930), with 5 being the highest and 1 being the lowest “relative” pitch level of a speaker's normalized pitch range.

**Table 1:** The five-scale tone letters and tone features of lexical tone inventories among four Chinese dialects.

**Dialects**	**Tone 1**	**Tone 2**	**Tone 3**	**Tone 4**	**Tone 5**	**Tone 6**	**Tone 7**
Chengdu	35	31	53	13			
	High-rising	Low-falling	High-falling	Low-rising			
Changsha	34	13	42	45	21	14	
	Mid-rising	Low-rising	High-falling	High-rising	Low-falling	Low-rising	
Fuzhou	44	51	32	21	231	23	55
	High-level	High-falling	Mid-falling	Low-falling	Low-peaking	Mid-short	High-short
Xiamen	44	24	53	21	22	32	44
	High-level	Mid-rising	High-falling	Low-falling	Low-level	Mid-short	High-short

*The underline indicates the short duration of the checked tone*.

In the current study, disyllabic prosodic words in four Chinese dialects were investigated to manifest the binary contrast of metrically weak and strong units, since two syllables could constitute the most natural and standard foot in Chinese (Feng, 2017). Overall, the present study aims to answer the following research questions: (a) Beyond the correlate of pitch, is the duration of disyllabic words sensitive to metrical prominence in Chinese dialects? (b) Are there cross-dialectal differences in duration and pitch realizations under the same metrical structure (Left-dominant: Changsha dialect vs. Chengdu dialect; Right-dominant: Fuzhou dialect vs. Xiamen dialect)? (c) Could the previously proposed metrical tone sandhi among these four Chinese dialects be validated by the fine-grained analysis of GCA?

Accordingly, we proposed three predictions based on the previous studies as follows: Hypothesis 1 (H1): In the left-dominant structure, a long-short duration pattern may be found, similar to the pattern of neutral tones in Mandarin. Besides, the right-dominant structure might exhibit a short-long duration pattern. Hypothesis 2 (H2): Given the diversity of Chinese dialects, the pitch and duration patterns within the same metrical structure might be generally similar, but not identical. Hypothesis 3 (H3): The statistical result of GCA might corroborate the previous impressionistic description of the metrical tone sandhi.

## Methods

### Participants

Five local participants in each dialect were recruited as the representative speakers of Changsha dialect (*M*_*age*_ = 62.00 yrs., *SD* = 6.36 yrs.; 2 females, 3 males), Chengdu dialect (*M*_*age*_ = 58.60 yrs., *SD* = 7.33 yrs.; 1 female, 4 males), Fuzhou dialect (*M*_*age*_ = 62.40 yrs., *SD* = 6.50 yrs.; 2 females, 3 males), and Xiamen dialect (*M*_*age*_ = 61.40 yrs., *SD* = 8.73 yrs.; 2 females, 3 males). In total, 20 participants took part in this experiment. Consistent with the traditional manner of in-depth field investigation, we only chose the participants aged 50 and older as representatives of each dialect. The reason is that the phonology of nowadays young people is often greatly influenced by Beijing Mandarin (Yao and Chang, 2016).

All participants were born and raised in the downtown of the local cities without the experience of traveling outside for over 6 months. According to the self-report, they only acquired their native dialect without the experience of other Chinese dialects or foreign languages. This effectively avoided potentially prosodic influences from other dialects/languages (Archibald, 2009). Besides, they did not self-report any speech disorder or hearing impairment. Before the elicitation task *via* text prompts, we confirmed that all participants could read the text of Chinese characters normally. After the experiment, each participant was paid the equivalent of 15 USD in local currency for their travel and time.

### Stimuli

The experimental stimuli, listed in Supplementary Table 1 (due to its overlength), were disyllabic prosodic words partially chosen from the word list (Guo, 2020) designed to investigate tone realizations from a cross-dialectal perspective. The lexical items were compound words including nouns, verbs, and adjectives, which were frequently spoken words [such as “工人” (worker)] across all the four dialects. Besides, we also selected some colloquial words in each dialect from previous studies as supplements, such as /ts^h^u53 ting53/ (rooftop) used in Xiamen dialect only (Chen, 1987). Therefore, in the current study, we used a mixed word list with both dialect-universal and dialect-specific words.

To make sure that the tone sandhi could be comprehensively analyzed, the disyllabic lexical items contained all the possible tonal combinations in each dialect, and five lexical items were chosen under each tonal combination as tokens (Supplementary Table 1). Since the number of lexical tones is different in the four dialects, the number of tonal combinations is also different. For instance, in the four-toned Chengdu dialect, the number of tonal combinations is 4^*^4 = 16, with a total of 16^*^5 = 80 lexical items. As for Changsha dialect, the total experimental items were 6^*^6^*^5 = 180 words. In Fuzhou and Xiamen dialects, however, the Tones 6, 7 belong to the checked tones (or “Ru Tones”). First, they are naturally shorter than other lexical tones (Tones 1–5) in terms of duration. Moreover, the checked tones are also different from other lexical tones with unique coda such as glottal stop /-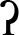/ or consonant stops (/-p/, /-t/, and /-k/). When they precede other lexical tones, its coda may drop which causes the compensatory lengthening of the duration (Chen and Norman, 1965). To control variables and make duration analysis more comparable, the checked tones (Tone 6 and Tone 7) in Fuzhou and Xiamen dialects were not included in the current study. Thus, there were 5^*^5^*^5 = 125 lexical items for both Fuzhou and Xiamen dialects.

### Recording Procedures

The recordings were conducted in quiet rooms located in Changsha, Chengdu, Fuzhou, and Xiamen city, respectively. To record high-quality audio samples, we used the cardioid microphone (AKG-C554L) connected to a USB audio interface (iCON4 nano VST). The recording for each dialect was conducted with a relatively low-level environmental noise (under 30 dB SPL). Before the formal recording, all the lexical items were shown to participants to familiarize them with the recording materials. Besides, a pilot investigation was carried out to confirm that five disyllabic prosodic words under each tonal combination showed the same tonal representation.

To control the pronunciation variables, a carrier sentence “这是__ (This is __)” was used before the target word. Participants were asked to produce the pre-target carrier sentence and the target word in a natural manner. All the target words were presented in a random order among participants. Specifically, participants could see the Chinese characters and their related lexical meanings of the target word on a laptop screen. Then, they spontaneously produced both the carrier and target word three times based on these prompts. All the participants of each dialect correctly uttered the target words, and the signals were saved in a WAV format with a sampling rate of 44.1 kHz.

After the collection of raw data, all the sound files were processed by the Praat (v.6.0.26) (Boersma and Weenink, 2021) on a PC laptop. Due to the occasionally poor sound quality (i.e., creaky voice) spoken by some participants, the pitch tracking might drop out throughout the syllable. To obtain more reliable results, only the best sound file with continuous pitch contours (exhibited in Praat) for each lexical item was included in the statistical analysis. In some cases, all the three recorded samples of the target word showed pitch-tracking failure, then we manually fixed the pitch tracking by the pitch tier function in Praat (Styler, 2011). In sum, there were 900 analyzed tokens in Changsha dialect (5 speakers × 180 words), 400 tokens in Chengdu dialect (5 speakers × 80 words), and 625 tokens in both Fuzhou dialect (5 speakers × 125 words) and Xiamen dialect (5 speakers × 125 words), with 2,550 analyzed tokens in total.

### Measurement and Data Analysis

The duration of each syllable was measured from the finals such as vowel (V), nasal rhyme (VN), and rhyme with glide (GV), since these different finals in Mandarin would not cause a significant duration difference (Wu and Kenstowicz, 2015). These finals were manually identified based on the spectrogram information in Praat, i.e., the onset and offset of the second formant (F2) within finals (Turk et al., 2006). Then, the raw duration was normalized for each participant with the z-score method according to Rose (1987). Since the duration is often skewed in distribution, the normalized duration was log-transformed as the dependent variable when entering the statistical models. In addition, to compare duration contrast among four dialects, we also calculated the σ(1) to σ(2) mean ratio of the absolute duration.

The fundamental frequency (F0) was extracted from each manually labeled syllable, in which 11 equal-distance points for the pitch trajectory were outputted. These F0 points were further checked and manually corrected for any “pitch-halving” or “pitch-doubling” errors which are detected when the determined F0 value is 20% higher or lower than the reference F0 value (Sun, 2002). Then, the raw F0 values (in Hz) by each participant were transformed into the logarithmic z-score values to eliminate individual differences in pitch range (Zhu, 2010).

All acoustic data were analyzed using R (R Core Team, 2020). To compare the duration between two consecutive syllables, a one-way ANOVA was conducted for each dialect. Moreover, linear mixed-effect models were conducted with the lme4 package (Bates et al., 2015). The *p*-values of fixed factors and their interaction were obtained with a type-II ANOVA using Wald chi-square tests *via* the car package (Fox and Weisberg, 2019). Furthermore, second-order orthogonal polynomials were built to compare three parameters of pitch contours (Mirman, 2014): the intercept term (i.e., overall pitch height), the first-order linear term (i.e., pitch slope), and the second-order quadratic term (i.e., pitch curvature).

The random slopes and intercept were incorporated in all models to make it generalizable across data maximally (Barr et al., 2013). Then, model comparisons were conducted to find out the best-fit model based on the Akaike information criterion (AIC) using the MuMIn package (Bartoń, 2022). When a significant main effect of a multilevel factor or a significant interaction effect was found, *post-hoc* pairwise comparisons were performed by using the lsmeans package (Lenth, 2016) with Tukey adjustment.

## Results

**Figure 2:**
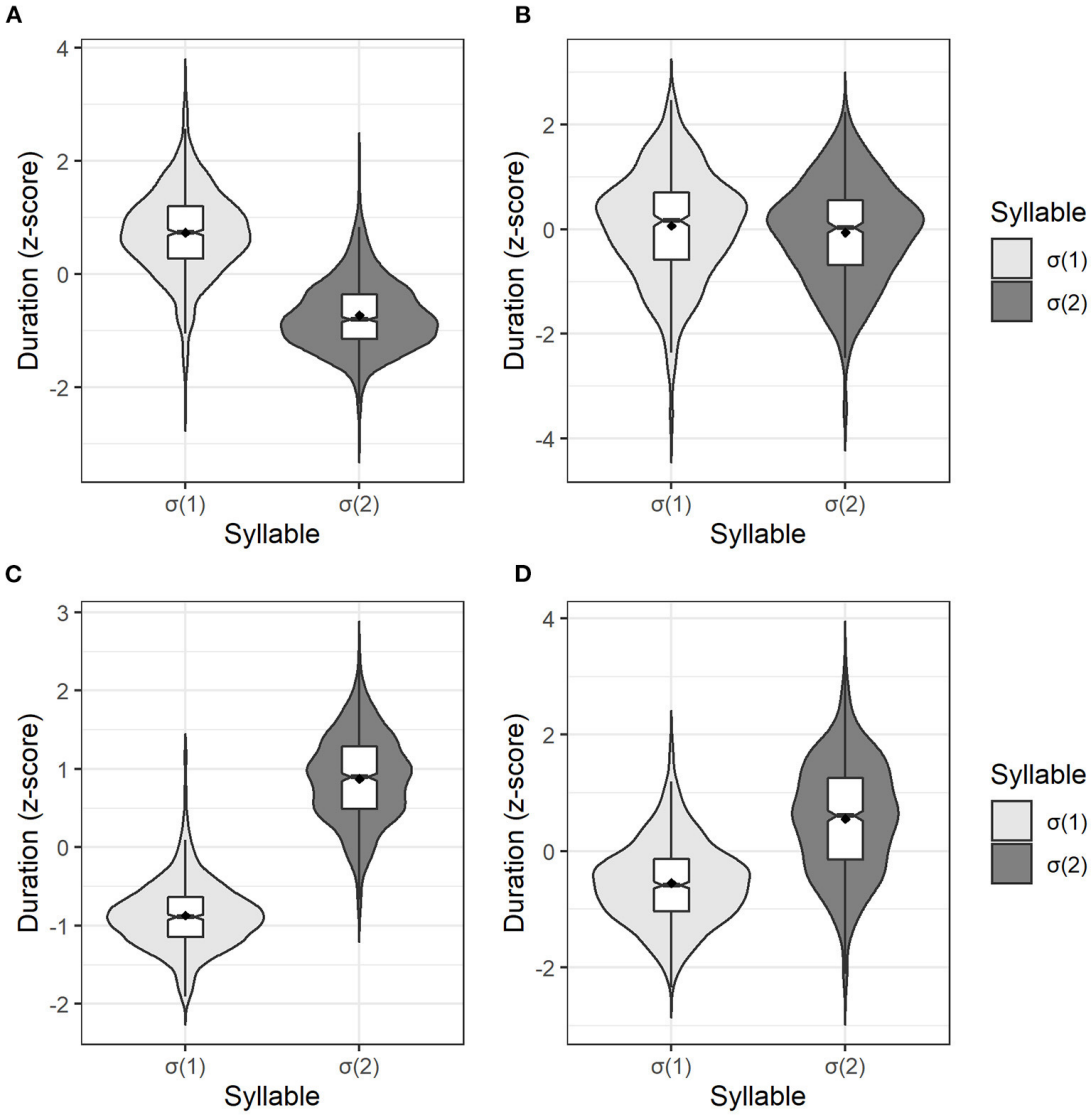
The normalized duration of σ(1) and σ(2) in disyllabic words of Changsha **(A)**, Chengdu **(B)**, Fuzhou **(C)**, and Xiamen dialect **(D)**.

**Figure 3:**
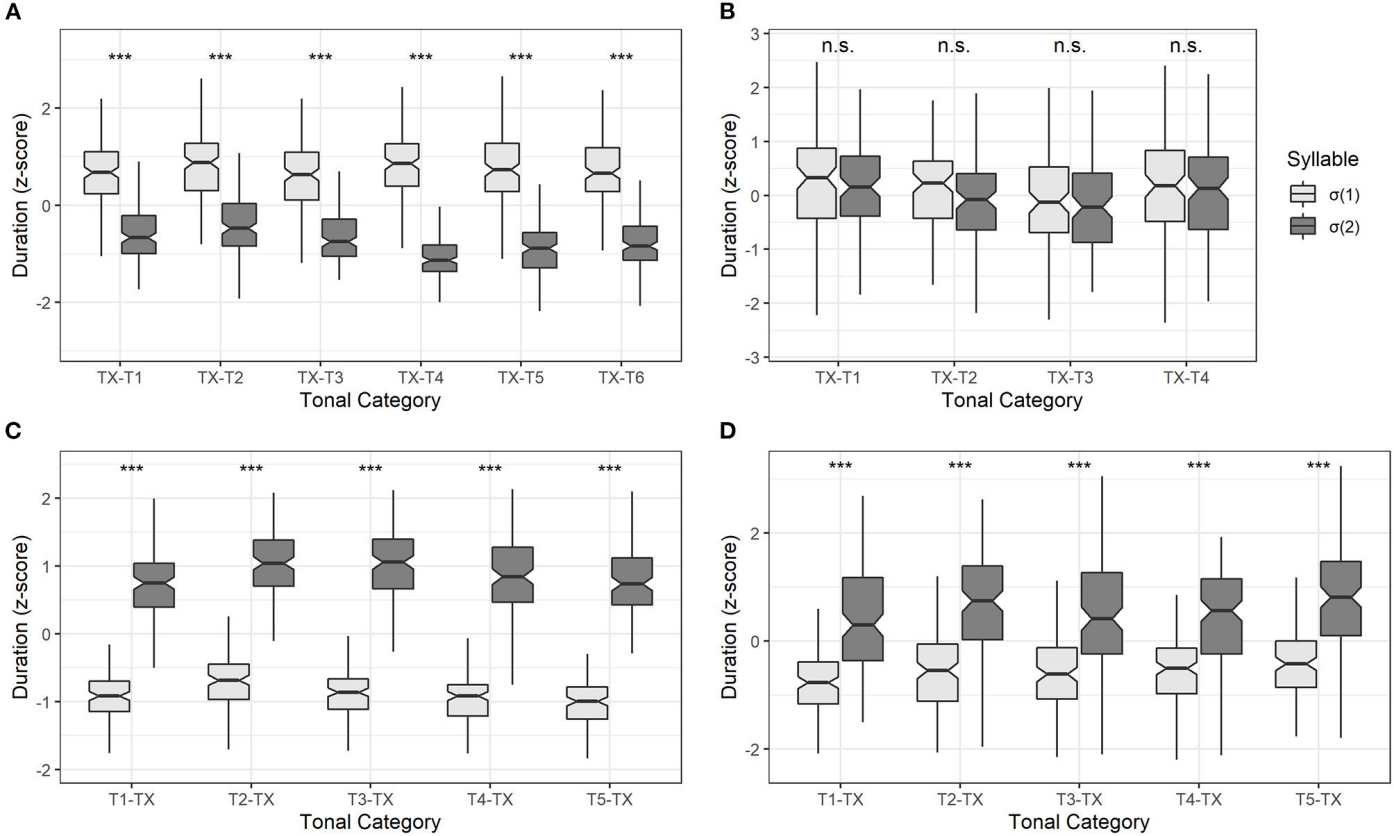
The normalized duration of σ(1) and σ(2) with different tonal categories in Changsha **(A)**, Chengdu **(B)**, Fuzhou **(C)**, and Xiamen dialect **(D)**. Asterisks (***) stand for *p* < 0.001. The “n.s.” stands for a *p*-value higher than 0.05.

Figure 2 shows the distribution of normalized duration of σ(1) and σ(2) in Four dialects, and Figure 3 further shows the normalized duration under different tonal categories classified by σ(2) (Changsha and Chengdu dialects) and σ(1) (Fuzhou and Xiamen dialect). The specific values from Figure 3 are listed in Table 2.

**Table 2:** The mean normalized duration (in z-score) and standard deviation (in bracket) with different tonal categories in σ(2) in Changsha and Chengdu dialects, whereas tonal categories in σ(1) in Fuzhou and Xiamen dialects.

**Tonal category**	**Changsha**		**Chengdu**		**Tonal category**	**Fuzhou**		**Xiamen**	
	**σ(1)**	**σ(2)**	**σ(1)**	**σ(1)**		**σ(1)**	**σ(2)**	**σ(1)**	**σ(2)**
TX-T1	0.68 (0.70)	−0.57 (0.57)	0.10 (1.15)	0.03 (1.04)	T1-TX	−0.92 (0.35)	0.73 (0.54)	−0.75 (0.63)	0.43 (0.92)
TX-T2	0.85 (0.75)	−0.41 (0.67)	0.13 (0.86)	−0.12 (0.93)	T2-TX	−0.70 (0.43)	1.05 (0.49)	−0.51 (0.81)	0.67 (0.92)
TX-T3	0.62 (0.74)	−0.67 (0.49)	−0.09 (1.04)	−0.20 (0.90)	T3-TX	−0.82 (0.48)	1.03 (0.52)	−0.56 (0.74)	0.46 (1.01)
TX-T4	0.80 (0.75)	−1.05 (0.51)	0.13 (1.06)	0.04 (0.97)	T4-TX	−0.92 (0.42)	0.80 (0.59)	−0.55 (0.65)	0.47 (0.85)
TX-T5	0.72 (0.84)	−0.88 (0.59)			T5-TX	−1.01 (0.37)	0.76 (0.51)	−0.39 (0.64)	0.73 (1.02)
TX-T6	0.71 (0.72)	−0.78 (0.61)							

*σ(1) = the initial syllable; σ(2) = the final syllable*.

### Left-Dominant Structure: Changsha Dialect

#### Duration Realization

The distribution of normalized duration of both syllables is shown in Figure 2A, indicating that the initial syllable σ(1) tended to be longer than the final syllable σ(2) in Changsha dialect. One-way ANOVA showed a significant difference in duration between two syllables [*F*_(1, 1, 798)_ = 2,033, *p* < 0.001]. Specifically, the σ(1) to σ(2) mean ratio of the absolute duration was about 1.53 (*SD* = 0.48) in Changsha dialect.

Furthermore, Table 2 shows the mean values and standard deviations of normalized durations in two syllables when the σ(2) carries different tonal categories. For example, TX-T1 stands for 6 tonal combinations: T1-T1, T2-T1, T3-T1, T4-T1, T5-T1, and T6-T1 (“T1” stands for “Tone 1,” etc. Abbreviations will be used below). As can be seen, all the mean normalized durations of σ(2) were negative values, while those of σ(1) were positive. Thus, compared with σ(1), the duration in σ(2) was phonetically reduced.

A linear mixed-effect regression model was constructed to test the normalized duration (logarithmic scale) difference of two syllables among the 6 tonal categories in σ(2). There were two fixed factors; one was *syllable* [σ(1) and σ(2)], and the other was *tonal category* (TX-T1, TX-T2, TX-T3, TX-T4, TX-T5, and TX-T6). The *participant* (5 individuals) and *word* (180 words) were included as the random factors. The model comparison only showed a significant main effect of *syllable* [χ^2^(1) = 12.91, *p* < 0.001], while the effect of *tonal category* [χ^2^(5) = 2.63, *p* = 0.756] and the interaction effect of *syllable* × *tonal category* [χ^2^(5) = 4.46, *p* = 0.486] failed to reach significance. These results indicated that the duration contrast was significant regardless of tonal categories in Changsha dialect (see Figure 3A).

#### Pitch Realization

The pitch realizations of all the tonal combinations in Changsha dialect are listed in Table 3. As can be seen, the sandhi form usually appeared in σ(2), while the σ(1) generally maintained the original pitch values (except for T3 [42] → [44]). In most cases, the σ(1) retained the underlying pitch form, while the σ(2) lost its original contour and became a level tone in Changsha dialect.

**Table 3:** The relative pitch values of lexical tones in Changsha disyllabic words.

**σ(1)/σ(2)**	**Tone1[34]**	**Tone2[13]**	**Tone3[42]**	**Tone4[45]**	**Tone5[21]**	**Tone6[14]**
Tone1[34]	[34.**33**]	[34.**33**]	[34.**44**]	[34.**44**]	[34.21]	[34.**44**]
Tone2[13]	[13.**33**]	[13.**33**]	[13.**44**]	[13.**44**]	[13.21]	[13.**44**]
Tone3[42]	[**44**.**33**]	[**44**.**33**]	[**44**.**44**]	[**44**.**44**]	[**44**.21]	[**44**.**44**]
Tone4[45]	[45.**33**]	[45.**33**]	[45.**44**]	[45.**44**]	[45.21]	[45.**44**]
Tone5[21]	[21.**33**]	[21.**33**]	[21.**44**]	[21.**44**]	[21.21]	[21.**44**]
Tone6[14]	[24.**33**]	[24.**33**]	[24.**44**]	[24.**44**]	[24.21]	[24.**44**]

*The dot “.” stands for syllable break, and numbers in bold indicate sandhi forms*.

**Figure 4:**
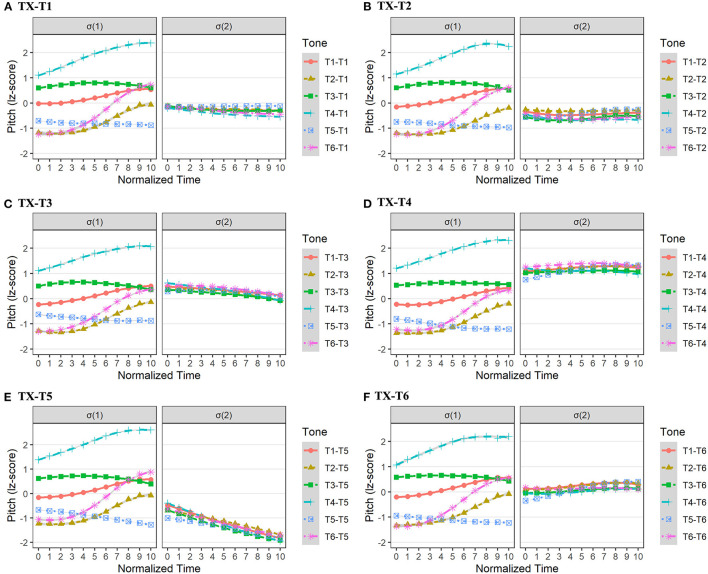
The pitch realizations of σ(1) and σ(2) in Changsha dialect with tonal combinations of TX-T1 **(A)**, TX-T2 **(B)**, TX-T3 **(C)**, TX-T4 **(D)**, TX-T5 **(E)**, and TX-T6 **(F)**.

In Changsha dialect, the sandhi form mainly emerged in the σ(2), such as T1[34] → [33], T2[13] → [33], T3[42] → [44], T4[45] → [44], and T6[14] → [44]. Only T5 in σ(2) was realized as an underlying form. The consistent trend of tonal processes was that the underlying tone in σ(2) lost its original contour, and was realized as a level tone. Moreover, the pitch value of these surface (level) tones was well below the highest pitch value of underlying rising tones. The pitch realizations in Changsha dialect are presented in Figure 4.

From Figure 4, we can see that the surface level tones in σ(2) were largely blended together regardless of the preceding tonal contexts (T1 to T6). To test the dependability of tonal aggregations in σ(2), we built 6 linear mixed regression models with second-order orthogonal polynomials. The fixed factor was *tonal context*, and the random factors were *participant* and *word*. We predicted that the different tonal contexts in σ(1) would not exert a significant influence on the intercept, slope, and curvature of target pitch contours in the σ(2).

For TX-T1 in Changsha dialect, *tonal context* showed no significant effect on both pitch intercept and curvature in σ(2) (*ps* > 0.05), but exerted a significant effect on pitch slope [χ^2^(5) = 12.43, *p* < 0.05]. However, *post-hoc* pairwise comparisons did not show a slope difference in the surface tones in σ(2) (*ps* > 0.05; Supplementary Table 2a). In addition, the *tonal context* in σ(1) exerted no significant effect on the pitch intercept, slope, and curvature in σ(2) of both TX-T2 and TX-T3 in Changsha dialect (*ps* > 0.05).

For TX-T4 in Changsha dialect, the *tonal context* showed no significant influence on the pitch intercept and curvature in σ(2) (*ps* > 0.05), but a significant effect on pitch slope [χ^2^(5) = 29.28, *p* < 0.001]. Specifically, the pitch contour in σ(2) of T5-T4 had a more rising trend than that of T1-T4, T3-T4, T4-T4, and T6-T4 (*ps* < 0.05; Supplementary Table 2b).

Moreover, for TX-T5 in Changsha dialect, the *tonal context* only showed a significant effect on the pitch slope in σ(2) [χ^2^(5) = 11.35, *p* < 0.05]. *Post-hoc* pairwise analysis suggested that the pitch contour in σ(2) of T5-T5 had a more moderate falling trend than that of T4-T5 (β = 0.94, *SE* = 0.31, *t* = 3.00, *p* < 0.05). Similarly, for TX-T6 in Changsha dialect, the *tonal context* only showed a significant influence on pitch slope [χ^2^(5) = 36.05, *p* < 0.001]. *Post-hoc* pairwise analysis indicated that the pitch contour in σ(2) of T5-T6 had a more rising trend than that under any other tonal contexts, *ps* < 0.01 (Supplementary Table 2c).

To conclude, the fine-grained analyses revealed that, although the preceding tonal context of T5 ([21]) mainly caused the subtly different pitch slope in σ(2), the majority of pitch height and curvature in σ(2), as indicated by the intercept and quadratic term, showed no significant differences under different tonal contexts. These results indicated that surface tones in σ(2) were largely overlapping level tones with similar pitch height in Changsha dialect.

### Left-Dominant Structure: Chengdu Dialect

#### Duration Realization

The distribution of normalized duration between σ(1) and σ(2) is shown in Figure 2B. The result of one-way ANOVA indicated that the normalized duration difference between σ(1) and σ(2) was non-significant [*F*_(1, 798)_ = 3.39, *p* = 0.066]. Moreover, the mean absolute duration ratio of σ(1) to σ(2) was 1.03 (*SD* = 0.15) in Chengdu dialect.

To investigate the duration difference of two syllables when the σ(2) carries different tonal categories, the mean normalized durations, and standard deviations are listed in Table 2. Due to the four-tone inventory of Chengdu dialect, TX-T1 represents all the tonal combinations such as T1-T1, T2-T1, T3-T1, and T4-T1.

A linear mixed-effect model was built to test the normalized duration (logarithmic scale) difference of two syllables among the 4 tonal categories in Chengdu dialect. The two fixed factors were *syllable* [σ(1) and σ(2)] and *tonal category* (TX-T1, TX-T2, TX-T3, and TX-T4), and the random factors were *participant* (5 individuals) and *word* (80 words). After model comparisons, neither the main effects of *syllable* [χ^2^(1) = 3.76, *p* = 0.053] and *tonal category* [χ^2^(3) = 4.91, *p* = 0.179], nor the interaction effect of *syllable* × *tonal category* reached significance [χ^2^(3) = 1.61, *p* = 0.658]. Therefore, the duration between σ(1) and σ(2) in Chengdu dialect was generally comparable among different tonal categories (see Figure 3B).

#### Pitch Realization

The pitch realizations of all the tonal combinations in Chengdu dialect are shown in Table 4. The sandhi forms appeared in both σ(1) and σ(2). To be specific, in σ(1), the surface tonal representation of T2 ([31]) was [33], and the surface form of T3 ([53]) was [45]. Furthermore, for the sandhi forms in σ(2), T1, T2 (in the tonal sequence of T2-T2), and T4 lost their underlying contours and became level tones on the surface. For example, T1 underwent from [35] to [33], T2 (when preceded by another T2) underwent from [31] to [33], and T4 underwent from [23] to [22].

**Table 4:** The relative pitch values of lexical tones in Chengdu disyllabic words.

**σ(1)/σ(2)**	**Tone1[35]**	**Tone2[31]**	**Tone3[53]**	**Tone4[23]**
Tone1[35]	[35.**33**]	[35.31]	[35.53]	[35.**22**]
Tone2[31]	[**33.33**]	[**33.33**]	[**33**.**31**]	[**33**.**22**]
Tone3[53]	[**45.33**]	[**45**.31]	[**45**.53]	[**45**.**22**]
Tone4[23]	[23.**33**]	[23.31]	[23. 53]	[23.**22**]

*The dot “.” indicates the syllable break, and numbers in bold indicate sandhi forms*.

**Figure 5:**
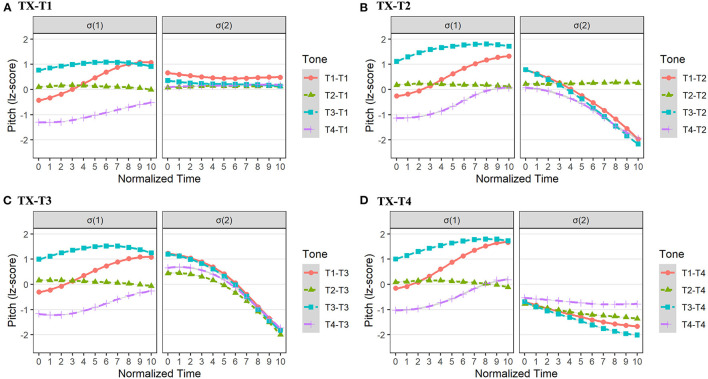
The pitch realizations of σ(1) and σ(2) in Chengdu dialect with tonal combinations of TX-T1 **(A)**, TX-T2 **(B)**, TX-T3 **(C)**, and TX-T4 **(D)**.

The pitch contours of different tonal combinations in Chengdu dialect are drawn in Figure 5. To verify the reliability of pitch values in Table 4, we built four linear mixed regression models with second-order polynomials to compare all the pitch contours in σ(2). The fixed factor of the models was the *tonal context* (T1-T4), and the random factors were *participant* and *word*.

For TX-T1 in Chengdu dialect, the model comparisons showed that the *tonal context* did not affect the pitch intercept, slope, or curvature in σ(2) significantly (*ps* > 0.05). Besides, for TX-T2 in Chengdu dialect, there was a significant main effect of *tonal context* on both pitch intercept [χ^2^(3) = 45.38, *p* < 0.001] and slope [χ^2^(3) = 47.02, *p* < 0.001] in σ(2). In terms of the pitch intercept, the *post-hoc* test indicated that the overall pitch height ([33]) in σ(2) of T2-T2 was higher than that ([31]) of T4-T2 (β = 0.60, *SE* = 0.17, *t* = 3.51, *p* < 0.01). As for the pitch slope in σ(2), the surface pitch contour ([33]) of T2-T2 had a flatter pitch contour than that ([31]) of T1-T2, T3-T2, and T4-T2 (*ps* < 0.001; Supplementary Table 3a).

For TX-T3 in Chengdu dialect, the results showed that the *tonal context* exerted impacts on both pitch intercept [χ^2^(3) = 8.26, *p* < 0.05], and pitch slope [χ^2^(3) = 14.72, *p* < 0.01]. *Post-hoc* pairwise comparisons on the pitch intercept were carried out, and results showed that the pitch height ([31]) in σ(2) of T2-T3 was lower than that ([53]) of T1-T3 (β = −0.60, *SE* = 0.21, *t* = −2.91, *p* < 0.05). Besides, the *post-hoc* test on the pitch slope indicated that the pitch contour ([31]) in σ(2) of T2-T3 had a more moderate falling trend than that in other tonal contexts (*ps* < 0.05; Supplementary Table 3b). Furthermore, for TX-T4 in Chengdu dialect, the model comparisons showed that the *tonal context* only exerted a significant influence on pitch slope in σ(2) [χ^2^(3) = 8.69, *p* < 0.05]. However, the *post-hoc* pairwise analysis did not show the pitch slope differences in σ(2) (*ps* > 0.05; Supplementary Table 3c).

In a nutshell, the results of GCA indicated that the surface form in σ(2) of T1 and T4 were realized as level tones (i.e., [33] and [22], respectively), and the tonal representation in σ(2) of T2-T2 was also a level tone [33] in Chengdu dialect. Furthermore, the other underlying tones of σ(2) were realized as falling tones with different pitch heights in Chengdu dialect.

### Right-Dominant Structure: Fuzhou Dialect

#### Duration Realization

The distribution of normalized duration between two syllables in Fuzhou dialect is shown in Figure 2C. The result of one-way ANOVA showed that the difference in duration between σ(1) and σ(2) was significant [*F*_(1, 1, 248)_ = 3,990, *p* < 0.001]. In addition, the mean σ(1) to σ(2) ratio of the absolute duration was around 0.57 (*SD* = 0.11) in Fuzhou dialect, indicating that the duration of σ(2) was significantly longer than that of σ(1).

Table 2 lists the mean values and standard deviations of the normalized duration in different tonal categories of σ(1) in Fuzhou dialect. The T1-TX represents tonal combinations of T1-T1, T1-T2, T1-T3, T1-T4, and T1-T5. Generally, the mean normalized durations of σ(1) were negative values, while those of σ(2) were positive values.

Then we built a linear mixed-effect model to test the difference in normalized duration (logarithmic scale) statistically. The fixed factors were *syllable* [σ(1) and σ(2)] and *tonal category* (T1-TX, T2-TX, T3-TX, T4-TX, and T5-TX). In addition, *participant* (5 individuals) and *word* (125 words) were included as the random factors. The model comparison only showed a significant main effect of *syllable* [χ^2^(1) = 12.69, *p* < 0.001]. Both the effect of *tonal category* [χ^2^(4) = 5.09, *p* = 0.279] and the interaction effect of *syllable* × *tonal category* [χ^2^(4) = 3.01, *p* = 0.556] were not found. Thus, the duration contrast between two syllables in Fuzhou dialect was significant across different tonal categories (see Figure 3C).

#### Pitch Realization

The surface tones of all the tonal combinations of disyllabic words in Fuzhou dialect are presented in Table 5. There is a noteworthy phenomenon in the pitch realization of Fuzhou dialect, that is, the surface tonal representations of certain tonal combinations are the same. Specifically, apart from checked tones, the pitch values of surface tones in T1-TX, T4-TX, and T5-TX were similar.

**Table 5:** The relative pitch values of lexical tones in Fuzhou disyllabic words.

**σ(1)/σ(2)**	**Tone1[44]**	**Tone2[51]**	**Tone3[32]**	**Tone4[21]**	**Tone5[231]**
Tone1[44]	[44.44]	[44.51]	[**52**.32]	[**52**.21]	[**52**.231]
Tone2[51]	[**44**.44]	[**32**.51]	[**32**.32]	[**21**.21]	[**21**.231]
Tone3[32]	[32.44]	[32.51]	[**24**.32]	[**44**.21]	[**44**.231]
Tone4[21]	[**44**.44]	[**44**.51]	[**52**.32]	[**52**.21]	[**52**.231]
Tone5[231]	[**44**.44]	[**44**.51]	[**52**.32]	[**52**.21]	[**52**.231]

*The dot “.” stands for syllable break, and numbers in bold indicate sandhi forms*.

When T1, T4, and T5 were in the σ(1), their sandhi forms could be described as (2a). The tone pattern was that the pitch in σ(2) was affected by the onset pitch height in σ(2). If the onset pitch height in σ(2) was low ([32]/[21]/[231]), the sandhi form of σ(1) was [52], ending with a low pitch value [2] accordingly. If the onset pitch height of σ(2) was high ([44]/[51]), then the sandhi form of σ(1) was [44], ending with a high pitch value [4]. In addition, the sandhi forms of T2 in σ(1) could be described as (2b). The pitch height of σ(1) was determined by the onset pitch height of σ(2) to a large degree. Overall, the direction of tonal assimilation was leftwards in Fuzhou dialect, the pitch of the right syllable σ(2) was likely to determine the pitch forms of σ(1) on the left.


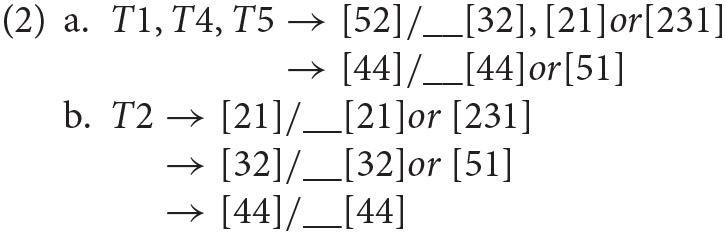


**Figure 6:**
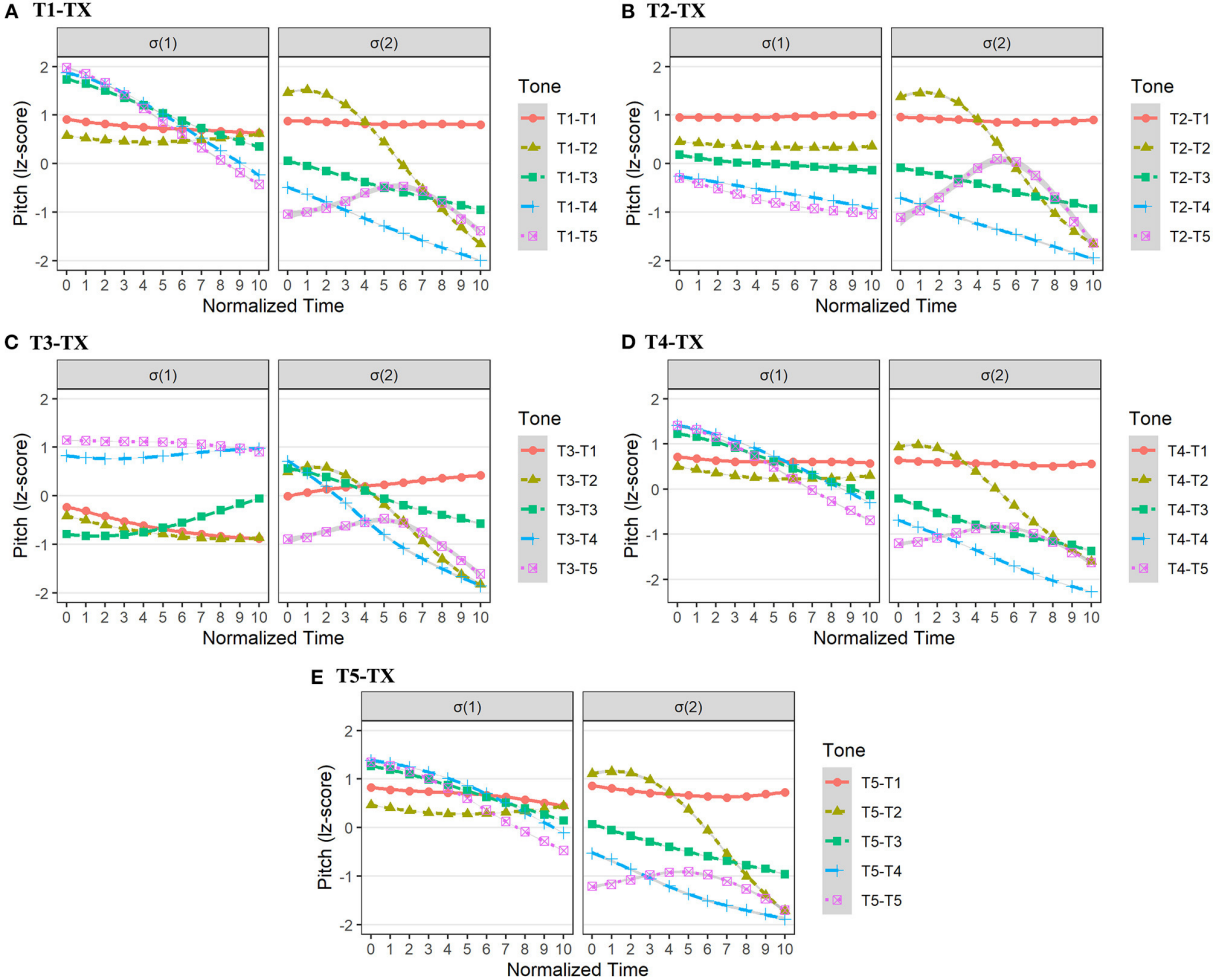
The pitch realizations of σ(1) and σ(2) in Fuzhou dialect with tonal combinations of T1-TX **(A)**, T2-TX **(B)**, T3-TX **(C)**, T4-TX **(D)**, and T5-TX **(E)**.

Figure 6 depicts the pitch contours of tonal combinations of T1-TX, T2-TX, T3-TX, T4-TX, and T5-TX in Fuzhou dialect. Then, five linear mixed-effect models with second-order orthogonal polynomials were constructed to compare the pitch of the σ(1). The fixed factor was the *tonal context*, and the random factors were *participant* and *word*. It is assumed that for T1, T4, and T5 in σ(1), their sandhi forms [52] and [44] might be different in terms of both pitch intercept and slope. Another possible result was that three sandhi forms of T2 (i.e., [21]/[32]/[44]) in σ(1) might be different in both pitch intercept and slope.

For T1-TX in Fuzhou dialect, the model comparison showed that pitch contours in σ(1) were different in terms of pitch slope [χ^2^(4) = 32.51, *p* < 0.001] and pitch curvature [χ^2^(4) = 17.43, *p* < 0.01]. *Post-hoc* pairwise analysis on pitch slope showed that the pitch contour ([44]) in σ(1) of T1-T1 and T1-T2 had a flatter trend than that ([52]) of T1-T3, T1-T4, and T1-T5 (*ps* < 0.01; Supplementary Table 4a).

Similarly, for T4-TX in Fuzhou dialect, the *tonal context* exerted a significant effect on both pitch slope [χ^2^(4) = 48.71, *p* < 0.001] and pitch curvature [χ^2^(4) = 20.21, *p* < 0.001] in σ(1). To be more specific, *post-hoc* pairwise comparisons on pitch slope showed that the pitch contour ([44]) in σ(1) of T4-T1 and T4-T2 had a flatter trend than that ([52]) of T4-T3, T4-T4, and T4-T5 (*ps* < 0.05; Supplementary Table 4b).

Moreover, for T5-TX in Fuzhou dialect, the main effects of the *tonal context* on pitch slope [χ^2^(4) = 20.38, *p* < 0.001] and pitch curvature [χ^2^(4) = 26.28, *p* < 0.001] were found again. *Post-hoc* pairwise comparisons on pitch slope were carried out (Supplementary Table 4c). Compared with T5-T3, T5-T4, and T5-T5, the pitch contour ([44]) in σ(1) of T5-T1 and T5-T2 was more significantly flatter (*ps* < 0.05).

In addition, the model comparison showed that T2's sandhi forms ([21]/[32]/[44]) in Fuzhou dialect differentiated from each other in both pitch intercept [χ^2^(4) = 45.28, *p* < 0.001] and pitch slope [χ^2^(4) = 16.11, *p* < 0.01]. The *post-hoc* pairwise analysis on pitch intercept in σ(1) was carried out (see Supplementary Table 4d). Results showed that compared with T2-T3, T2-T4, and T2-T5, the pitch height in σ(1) of T2-T1 ([44]) was significantly higher (*ps* < 0.01).

To conclude, in Fuzhou dialect, statistical results suggested that two sandhi forms ([52]/[44]) of T1, T4, and T5 in σ(1) were conditioned by following tonal contexts mainly in terms of pitch slope, rather than pitch height. Furthermore, the tonal representation of T2 ([21]/[32]/[44]) in the surface was strictly modulated by the pitch height of the following tonal contexts.

### Right-Dominant Structure: Xiamen Dialect

#### Duration Realization

The distribution of normalized duration in Xiamen dialect is drawn in Figure 2D. The result of one-way ANOVA showed that the difference in duration in two syllabic positions was significant [*F*_(1, 1, 248)_ = 542.60, *p* < 0.001]. Besides, the mean duration ratio in Xiamen dialect was about 0.83 (*SD* = 0.14).

Divided by tone categories in σ(1), the mean normalized durations and standard deviations of two syllables are listed in Table 2. The mean normalized durations of σ(1) were negative values, while those of σ(2) were positive values. Furthermore, the standard deviations in σ(2) were greater than those in σ(1) in Xiamen dialect.

A linear mixed-effect model was constructed to test the duration pattern (logarithmic scale) across 5 tonal categories. The *syllable* [σ(1) and σ(2)], *tonal category* (T1-TX, T2-TX, T3-TX, T4-TX, and T5-TX) were set as the fixed factors, and the *participant* (5 individuals) and *word* (125 words) were included as the random factors. The result showed a significant effect of *syllable* [χ^2^(1) = 31.25, *p* < 0.001]. However, *tonal category* did not show an effect on duration [χ^2^(4) = 7.29, *p* = 0.121]. The interaction effect of *syllable* × *tonal category* was not found [χ^2^(4) = 9.34, *p* =0.053]. Results indicated that the duration difference between σ(1) and σ(2) was significant among tonal categories in Xiamen dialect (see Figure 3D).

#### Pitch Realization

The pitch realizations of all tonal combinations in Xiamen dialect are listed in Table 6. As can be seen, the σ(2) maintained its underlying pitch form, yet the underlying tone in σ(1) was realized as its sandhi form, similar to that in Fuzhou dialect.

**Table 6:** The relative pitch values of lexical tones in Xiamen disyllabic words.

**σ(1)/σ(2)**	**Tone1[44]**	**Tone2[24]**	**Tone3[53]**	**Tone4[21]**	**Tone5[22]**
Tone1[44]	[**33**.44]	[**33**.24]	[**33**.53]	[**33**.21]	[**33**.22]
Tone2[24]	[**33**.44]	[**33**.24]	[**33**.53]	[**33**.21]	[**33**.22]
Tone3[53]	[**44**.44]	[**44**.24]	[**44**.53]	[**44**.21]	[**44**.22]
Tone4[21]	[**53**.44]	[**53**.24]	[**53**.53]	[**53**.21]	[**53**.22]
Tone5[22]	[**21**.44]	[**21**.24]	[**21**.53]	[**21**.21]	[**21**.22]

*The dot “.” stands for syllable break, and numbers in bold indicate sandhi forms*.

**Figure 7:**
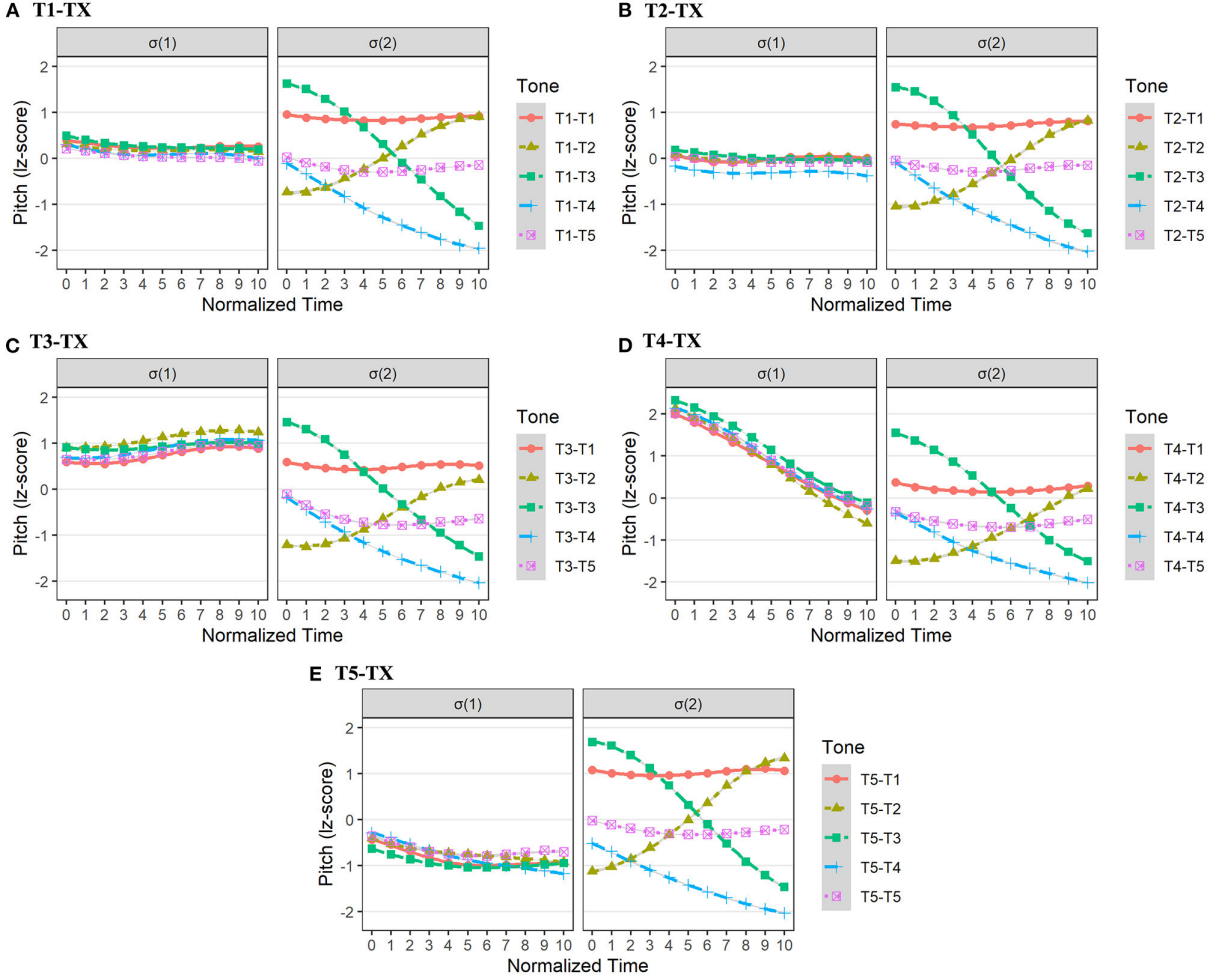
The pitch realizations of σ(1) and σ(2) in Xiamen dialect with tonal combinations of T1-TX **(A)**, T2-TX **(B)**, T3-TX **(C)**, T4-TX **(D)**, and T5-TX **(E)**.

The sandhi form ([33]) of T1 in σ(1) was lower than its underlying form ([44]). For T2 in Xiamen dialect, its underlying pitch form ([24]) in σ(1) lost rising contour and became level tones ([33]). Likewise, the sandhi form ([53]) of T3 was also a level tone ([44]). Therefore, T1, T2, and T3 probably underwent tonal neutralization, since their surface tones in σ(1) generally approached the mid-level tone [33] (see Figure 7). In addition, the sandhi forms of T4 and T5 in σ(1) were falling tones, [53] and [21], respectively. Then, 5 second-order orthogonal polynomial models were built to compare pitch forms in σ(1) across all tonal contexts (T1-TX, T2-TX, T3-TX, T4-TX, and T5-TX). The fixed factor of each model was *tonal context*, and the random factors were *participant* and *word*. It was presumed that *tonal context* exerted no significant effect on the pitch intercept, pitch slope, or pitch curvature of surface tones in σ(1).

For T1-TX in Xiamen dialect, the model comparisons showed that *tonal context* did not affect pitch intercept, slope, and curvature (*ps* > 0.05). Besides, the *tonal context* did not affect the pitch intercept, slope, and curvature of σ(1) in T2-TX either (*ps* > 0.05). Thus, the sandhi form [33] of underlying T1 ([44]) or T2 ([24]) was stable irrespective of the following tonal contexts in σ(2). Likewise, we did not find the main effect of *tonal context* on the pitch intercept, slope, and curvature in the σ(1) of T3-TX (*ps* > 0.05), suggesting that the sandhi form ([44]) was not affected by the following lexical tones in σ(2).

Moreover, the results of T4-TX and T5-TX also met our expectations, the *tonal context* exerted no impact on the intercept, slope, and curvature of pitch forms in σ(1) (*ps* > 0.05). Results indicated that the sandhi form of T4 in σ(1) was uniform, free from the influence of following tonal contexts, and so was T5.

To conclude, results indicated that in Xiamen dialect, the surface tone in σ(1) showed a uniform representation for each underlying tone, regardless of the tonal contexts in σ(2). Especially for T1, T2, and T3, they lowered pitch heights and lost underlying contours.

## Discussion

### The Limited Role of Duration in the Manifestation of Metrical Structure in Chinese Dialects

Generally speaking, the duration could be used as a phonetic cue measuring the syllable weight, since a longer duration might indicate a heavier syllable weight (Maddieson, 1993; Hubbard, 1994). Especially in the weight-sensitive languages, the heavy syllables carrying two morae exhibit a longer duration than the monomoraic light syllables, and also are easier to attract the metrical stress (Selkirk, 1982; McCarthy and Prince, 1994). The relation between the syllable weight and the word stress could be summarized as the “weight to stress principle” (Prince, 1990). The present study revealed the diversity of duration patterns between two types of metrical structure as shown in four Chinese dialects, and the results were discussed as follows.

According to our prediction (H1), the left-dominant structure might exhibit a long-short pattern in duration. Yet, our finding does not support the H1, since although the duration between two syllables was significantly different in Changsha dialect, that was evenly arranged in Chengdu dialect. Specifically, in Changsha dialect, the duration in σ(2) was significantly shorter than that in σ(1) regardless of tonal categories. This duration pattern was supported by the impressionistic description according to Zhong (2003). The mean duration ratio in Changsha dialect was about 1.53: 1, approaching the ratio (1.7: 1) of the neutral-tone words in Mandarin (Chen and Xu, 2006). It is worth noting that lexical items in our study were daily spoken words that did not belong to the category of the neutral-tone words used in a small number of Mandarin words (about 7% according to Li, 1981). However, the disyllabic words in Chengdu dialect exhibited comparable duration between two syllables (with a ratio of 1.03: 1), which pointed to the absence of a phonetic reduction in σ(2). Thus, the syllable weight indicated by duration was quite evenly matched in disyllabic words of Chengdu dialect. Given the left-dominant structure in Chengdu dialect according to Qin (2012), the duration may not act as the phonetic correlate of metrical prominence robustly. Potentially, the difference in duration pattern in these two dialects could be attributed to the dialect differences, since Changsha and Chengdu dialects belong to the Xiang dialect group and the Mandarin supergroup, respectively (see Figure 1A). It was reported that disyllabic words in the Yiyang dialect (Xiang dialect group) also showed a long-short duration pattern (Xia, 2018). More cross-dialectal research is needed to demonstrate the diverse duration patterns of the left-dominant metrical structure.

On the contrary, a short-long duration pattern was consistently found under the right-dominant structure of both Fuzhou and Xiamen dialects. Such duration pattern might serve as the robust phonetic evidence for the right-dominant structure, indicating that Fuzhou and Xiamen dialects were weight-sensitive dialects. This finding is consistent with our prediction (H1) for the right-dominant structure, and is also in line with the “Iambic/Trochaic Law” (Hayes, 1995), suggesting that a short-long duration pattern commonly exists in the iamb. Although Fuzhou and Xiamen dialects shared a similar duration pattern, the duration ratio difference between two syllables was not exactly comparable in these two dialects. To be more specific, the duration contrast between σ(1) and σ(2) in Fuzhou dialect (a ratio of 0.57: 1) was generally greater than that in Xiamen dialect (a ratio of 0.83: 1). When focusing on the duration contrast under different tonal categories, as shown in Table 2, we could also observe a greater duration difference between σ(1) and σ(2) in Fuzhou dialect. A deeper understanding of this phonetic difference in the right-dominant structure requires further investigation.

In general, duration patterns in three out of four dialects corresponded with the underlying metrical structure. Thus, the metrical prominence in disyllabic words of Chinese dialects might not always be manifested as a longer duration. Such finding only supported our prediction (H1) regarding the right-dominant structure. In addition, consistent with the “Iambic/Trochaic Law” (Hayes, 1995), the duration pattern could reliably reflect the “relative relation” (i.e., light or heavy) of the syllable weight in the right-dominant structure of Chinese dialects. Future research needs to be carried out to investigate whether there is a final-lengthening effect on duration realization by putting the target words at different prosodic positions of the carrier sentence.

### The Robust Role of Pitch in the Manifestation of Metrical Structure in Chinese Dialects

It was proposed that pitch realizations between metrically weak and strong positions in Chinese have opposite tendencies. One is that, in the metrically weak unit such as σ(w), the lexical tone tends to lose its underlying contour (Chen and Xu, 2006), or further undergoes tonal alternation (Yue-Hashimoto, 1987; Chen, 2000); The other is that the syllable with more prosodic strength [σ(s)] would fully exhibit the underlying tonal representation and keep its original tone features (Kochanski et al., 2003; Zeng and Niu, 2006). The current study investigated the pitch realizations of four Chinese dialects by using the second-order orthogonal polynomials models to compare the pitch intercept, slope, and curvature.

Table 7 shows tonal alternations in the metrically weak position across the four dialects. Specifically, there were generally three trends of tonal changes according to underlying pitch contours: (1) The underlying rising tone was generally realized as a level tone on the surface (i.e., [44]/[33]/[22]). Moreover, the pitch height of the surface level tone was below the peak pitch of the underlying form, except for Fuzhou dialect (no underlying rising tones in its tone inventory); (2) For underlying level tones, they were prone to change into falling tones with similar pitch height. Note that there are no underlying level tones in the tone inventories of Chengdu and Changsha dialects; (3) The underlying falling tones tended to be realized as level tones with similar pitch heights (i.e., [33]/[44]), or sometimes be realized with original falling contour but different pitch heights. In summary, a notably consistent tonal pattern was that underlying contour tones (falling or rising) were generally realized as level tones on the surface. In other words, the level tone was a more common surface representation in the metrically weak position.

**Table 7:** The tonal alternations in the metrically weak position across the four dialects.

**Underlying**	**Changsha**	**Chengdu**	**Fuzhou**	**Xiamen**
**contour**				
Rising	[45] → [44];			
	[34] → [33]	[35] → [33]		[24] → [33]
	[14] → [44];	[23] → [22]		
	[13] → [33]			
Level			[44] → [52]	[44] → [33]
				[22] → [21]
Falling	[42] → [44]	[31] → [33]	[51] → [21], [32] or [44]	[53] → [44]
			[32] → [24] or [44]	[21] → [53]
			[21] → [52] or [44]	

Moreover, it should be noted that these surface tones were sometimes context-dependent in the four dialects. In other words, the tone sandhi in a metrically weak position might be conditioned by the preceding/following tonal context (of metrically strong position). For instance, the pitch realizations of T2-T2 in Chengdu dialect and most tonal alternations in Fuzhou dialect were contextually conditioned. Specifically, in Chengdu dialect, the surface representation of T2 in σ(2) was realized as [33] only when preceded by another T2 in sequence (see Figure 5). Likewise, the surface pitch form of T2 in σ(1) was decided by the following tonal context in Fuzhou dialect (see Figure 6). These tonal changes are overall comparable to the neutral tones in Mandarin, where tone deletion occurs, leaving a vacant tone-bearing unit in the target position which awaits a proper pitch realization from the preceding tonal context (Wang, 2008). Likewise, the metrical weak unit might contain a single mora. Additionally, in Changsha and Xiamen dialects, the surface form in the metrically weak position of each underlying tone was uniform, regardless of tonal contexts (see Figures 4, 7). Besides, the underlying T1 and T4 in Chengdu dialect were also free from the contextual effect, realized as [33] and [22] respectively. The contextual dependency in the four dialects could be summarized as (3).


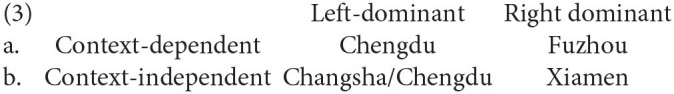


On the contrary, the metrically strong units generally maintained their underlying pitch forms. This indicates they might have two morae that could bear contour tones. It should be noted that there were two exceptions, namely the T2/T3 in Chengdu dialect and T3 in Changsha dialect. In Chengdu dialect, the tonal alternations in σ(1) showed that T2 ([31]) and T3 ([53]) underwent [31] → [33] and [53] → [45], respectively. Many other factors might influence the surface form of lexical tones. For example, it has been generally deemed that the tonal merger (or simplification) is mainstream in tonal development in contemporary Chinese dialects (Pan, 1982). One possibility is that the exceptions in Chengdu dialects were triggered by tonal mergers, and that all surface tones of T3-TX approached T1([35])-TX. This kind of tonal merger could be supported by the case of Fuzhou dialect. In Fuzhou dialect, the surface tones of two syllables among T1-TX, T4-TX, and T5-TX were identical (see Table 5), indicating a completed tonal merger. However, the reasons why underlying tones changed in σ(1) of T2 in Chengdu dialect and T3 in Changsha dialect are still unknown and need more future studies to find out.

In a nutshell, despite a few exceptions, the four Chinese dialects we investigated exhibited consistent tonal representations of a level pitch contour in the metrically weak position while the underlying pitch form in a strong position. Therefore, this finding corroborated many case studies across Chinese dialects (Yue-Hashimoto, 1987; Chen, 2000), and the above tonal representations were generally confirmed by the fine-grained analysis (GCA) as we predicted (H3). Furthermore, this surface tone of the metrically weak position under the same metrical structure might be classified into context-independent and context-dependent types. These different types also supported our prediction (H2) that the specific pitch realization might be not identical within a certain metical pattern.

### The “Metrical Tone Sandhi”: The Interaction Between Duration and Pitch Realizations

To date, although the tone sandhi in the metrically weak unit has been termed as the “metrical tone sandhi” (Zeng and Niu, 2006), many puzzles are still lingering. For instance, it is still unclear whether metrically tonal alternation is related to the duration pattern. We also wonder whether underlying tone would be realized as different surface forms according to different prosodic units. In the present study, we examined four Chinese dialects by drawing on the phonetic parameters of both duration and pitch, which offered a valuable chance to analyze their interaction. The “metrical tone sandhi” among the four Chinese dialects would be discussed below.

First, in Changsha dialect, the metrical structure belongs to the strong-weak pattern, exemplified by the shorter duration and surface level tones in σ(2). In terms of duration, the duration ratio in Changsha disyllabic words (1.53: 1) is close to that in Mandarin neutral-toned words (1.70: 1; Chen and Xu, 2006). We could assume that the σ(2) in Changsha dialect is monomoraic like the neutral-toned syllable in Mandarin (Duanmu, 1993), while the σ(1) is the heavy syllable containing two morae. Based on this pattern [σ(μμ) σ(μ)], the surface pitch realization of underlying T1, T2, T4, and T6 could be realized as (4) with T1 as the preceding context. Thus, the bimoraic rime of σ(1) in Changsha dialect could bear the underlying contour, while the surface tone of σ(2) could be realized as the level tone probably due to the limited capacity of tone bearing.


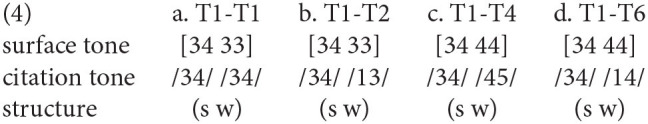


As for the disyllabic words in Chengdu dialect, the duration between two syllables is evenly arranged. The metrical pattern might be a syllabic trochee [σ(s) σ(w)]. Like Changsha dialect, the pitch contour of σ(2) in Chengdu dialect also undergoes a similar phonetic reduction, resulting in a level tone as the surface representation. For instance, the surface tone of T1 and T4 in σ(2) could be described as (5) when preceded by T1. Although the duration is not a phonetic correlate of the underlying metrical structure in Chengdu dialect, the strong-weak metrical pattern could be manifested by this tonal alternation in σ(2) (Qin, 2012).


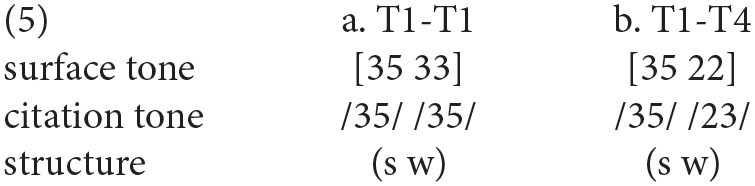


Then, for Fuzhou dialect, the duration in σ(2) is significantly longer than that in σ(1), indicating that σ(1) and σ(2) belong to the light and heavy syllables, respectively [σ(μ) σ(μμ)]. Under this circumstance, the underlying T1, T2, T4, and T5 in σ(1) lose their original tone features and are assimilated by the following tonal contexts. These tonal alternations could be illustrated as (6) with T1 as the following tonal context. The tonal processes of tone deletion in σ(1) and leftward tone spreading from σ(2) could be seen in Fuzhou dialect.


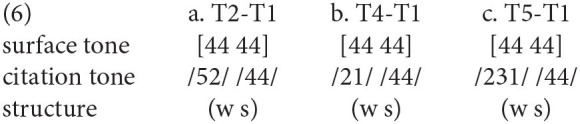


Similarly, the duration in σ(2) is also longer than the σ(1) counterpart in Xiamen dialect. Thus, the syllable weight between two syllables in Xiamen dialect is different. Given this pattern of syllable weight [σ(μ) σ(μμ)], the tonal neutralization occurs in the σ(1) in terms of underlying T1, T2, and T3. When these lexical tones are followed by T1, the tone sandhi could be seen as (7). The tonal neutralization at σ(1) could be attributed to the limited tone-bearing capacity in the monomoraic syllable. Based on the duration and pitch realizations, we might confirm the metrically weak-strong structure in Xiamen dialect.


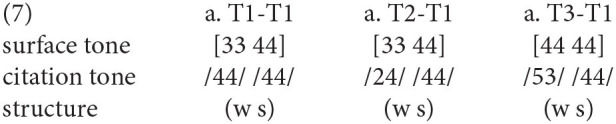


From the cross-dialectal perspective, the tonal process in the left-dominant structure contains contour reduction, apart from rightward tone spreading (Zhang, 2007). In the right-dominant, both leftward tone spreading and tonal neutralization could occur in the surface tonal representation. Although the above tonal processes are manifested in different manners, the core driving force is the underlying metrical structure in these four dialects. That is, the metrically weak syllable undergoes tone sandhi, while the strong syllable, where the metrical stress lies, is fully realized as the underlying pitch form (Duanmu, 1993; Chen, 2000; Zeng and Niu, 2006). In general, “metrical tone sandhi” enlightens us re-think the interaction between duration and pitch realizations in Chinese dialects. More cross-dialectal research, however, is needed to explore the fundamental effect of metrical structure on the tone sandhi more clearly.

Meanwhile, it is noteworthy that not all the tone sandhi in the four Chinese dialects could be interpreted as the “metrical tone sandhi.” For instance, the pitch change of the σ(1) in Chengdu dialect and the T4 ([21] → [53]) in Xiamen dialect are beyond the phenomenon of the “metrical tone sandhi.” To some extent, it might be more appropriate to regard them as dialect-specific tonal alternations.

## Conclusion

The current study presented the diverse phonetic realizations under two metrical structures across four Chinese dialects. Specifically, we examined the duration and pitch realizations of disyllabic prosodic words in Changsha and Chengdu dialects under the left-dominant structure, and in Fuzhou and Xiamen dialects under the right-dominant structure.

The results of cross-dialectal comparisons indicated that the duration patterns in four Chinese dialects were not always sensitive to different metrical structures, given that the duration contrast in Chengdu dialect was not significant. Therefore, the phonetic correlate of duration alone did not play a universal role in manifesting the metrical prominence. Moreover, the GCA was adopted to examine the pitch realization in the metrically weak position. The general tendency was that the main surface form in the prosodic weak element became level tones (sometimes falling tones). Compared with duration realization, pitch realization might be more robust as an indicator of metrically binary contrast in Chinese dialects. Furthermore, there might be interactions between duration and pitch realizations in Chinese dialects, thus the nature of “metrical tone sandhi” could unfold more clearly when combining pitch realization with the duration pattern.

## Data Availability Statement

The raw data supporting the conclusions of this article will be made available by the authors, without undue reservation.

## Ethics Statement

The studies involving human participants were reviewed and approved by Hunan University, School of Foreign Languages. The patients/participants provided their written informed consent to participate in this study.

## Author Contributions

CG and FC conceived and designed the study, participated in the statistical analysis, interpreted the data, and wrote the first draft of the manuscript. CG collected the data. Both authors contributed to and have approved the final manuscript.

## Funding

This study was supported by the MOE (Ministry of Education of China) Youth Fund Project of Humanities and Social Sciences Research (Grant No. 21YJC740015).

## Conflict of Interest

The authors declare that the research was conducted in the absence of any commercial or financial relationships that could be construed as a potential conflict of interest.

## Publisher's Note

All claims expressed in this article are solely those of the authors and do not necessarily represent those of their affiliated organizations, or those of the publisher, the editors and the reviewers. Any product that may be evaluated in this article, or claim that may be made by its manufacturer, is not guaranteed or endorsed by the publisher.
