# Supplementary material for: Phonetic Realizations of Metrical Structure in Tone Languages: Evidence From Chinese Dialects
Source: Front Psychol. 2022 Jul 13;13:945973. doi: 10.3389/fpsyg.2022.945973 (PMC9326321; doi:10.3389/fpsyg.2022.945973)
Supplement: Supplementary file 1 [file Data_Sheet_1.docx]

Supplementary Material

## Supplementary Table 1

**Table 1a** The disyllabic words of Changsha dialect

| T1-T1 | T2-T1 | T3-T1 | T4-T1 | T5-T1 | T6-T1 |
| --- | --- | --- | --- | --- | --- |
| 鸳鸯/yan jan/  Mandarin duck | 南瓜/lan kua/  pumpkin | 耳朵/ɤ to/  ear | 菜刀/tsʰai tau/  kitchen knife | 大衣/tai i/  coat | 国家/kuə tɕia/  country |
| 冬瓜/tən kua/  White gourd | 泥巴/ȵi pa/  mud | 嘴巴/tsei pa/  mouth | 亲家/tɕʰin ka/  relatives | 豆浆/təu tɕian/  soybean milk | 白天/pɤ tʰian/  daylight |
| 西瓜/ɕi kua/  watermelon | 棉花/miẽ fa/  cotton | 祖宗/tso səu/  ancestor | 桂花/kuei fa/  osmanthus | 大方/tai fan/  generous | 辣椒/la tɕiau/  pepper |
| 香蕉/ɕian tɕiau/  banana | 枇杷/pi pa/  loquat | 火车/xo tsʰɤ/  train | 杜鹃/təu tɕyan/  cuckoo | 汗衫/xan san/  shirt | 雪花/ɕie fa/  snowflake |
| 飞机/fei tɕi/  plane | 蜈蚣/u kən/  centipede | 马蜂/ma xoŋ/  wasp | 扫把/sau pa/  besom | 下巴/xa pa/  jaw | 作家/tso tɕia/  writer |
| T1-T2 | T2-T2 | T3-T2 | T4-T2 | T5-T2 | T6-T2 |
| 高粱/kau lian/  sorghum | 爷娘/ia ȵiaŋ/  parents | 母牛/mo ȵiəu/  cow | 画眉/fa mei/  thrush | 链常/liẽ tsan/  usually | 核桃/xɤ tʰau/  nut |
| 工人/kən zən/  worker | 眉毛/mei mau/  eyebrow | 海棠/xai tan/  begonia | 算盘/sõ põ/  abacus | 面条/miẽ tiau/  noodle | 客人/kʰɤ zən/  guest |
| 鹌鹑/ŋan ɕyn/  quail | 便宜/piẽ i/  cheap | 语言/y jan/  langugae | 汽油/tɕʰi iəu/  gasoline | 薄荷/po xo/  mint | 发型/fa xin/  haircut |
| 槟榔/pin lan/  areca-nut | 洋油/jan iəu/  kerosene | 枕头/tsən təu/  pillow | 太阳/tʰai ian/  sun | 外行/wai xan/  layman | 腊梅/la me/  wintersweet |
| 蜗牛/o ȵiəu/  snail | 明年/min ȵiẽ/  next year | 主人/tɕy zən/  master | 自然/tsɿ yan/  nature | 赚头/tɕyan təu/  profit | 轴承/tsou tsən/  bearing |
| T1-T3 | T2-T3 | T3-T3 | T4-T3 | T5-T3 | T6-T3 |
| 鸡眼/tɕi ŋan/  corn | 肥皂/fei tsau/  soap | 好歹/xau tai/  whatever | 戒指/kai tsɿ/  ring | 道理/tau li/  principle | 石榴/sɿ liəu/  pomegranate |
| 肩膀/tɕiẽ pan/  shoulder | 来往/lai wan/  contact | 腼腆/miẽ tiẽ/  shy | 战友/tsɤ̃ jəu/  comrade | 大米/tai mi/  rice | 读者/təu tsɤ/  reader |
| 开水/kʰai ɕyei/  boiled water | 柴火/tsai xo/  firewood | 讲法/kan fa/  statement | 信纸/ɕin tsɿ/  letter paper | 豆腐/təu fu/  tofu | 屋顶/wu tin/  roof |
| 真理/tsən li/  truth | 朋友/pən iəu/  friend | 左手/tso səu/  left hand | 户口/fu kʰəu/  residence | 右手/iəu səu/  right hand | 喫酒/tɕʰia tɕiəu/  drinking beer |
| 鹦鹉/in u/  parrot | 人手/zən səu/  manpower | 懵懂/mən tʰən/  ignorant | 电影/tiẽ in/  movie | 豆粉/təu fən/  bean flour | 查理/tsa li/  Charlie |
| T1-T4 | T2-T4 | T3-T4 | T4-T4 | T5-T4 | T6-T4 |
| 甘蔗/kan tsə/ | 玫瑰/mei kuei/ | 拐棍/kuai kuən/ | 故意/ku i/ | 地道/ti tau/ | 荸荠/pu tɕi / |
| sugarcane | rose | sticker | on purpose | tunnel | chufa |
| 家具/tɕia tɕy/ | 凉快/lian kʰuai/ | 买卖/mai mai/ | 自动/tsɿ toŋ/ | 饭铺/fan pʰu/ | 别个/pie ko/ |
| furniture | cool | bargain | automatic | restaurant | other pople |
| 铺盖/pʰu kai/ | 和气/xo tɕʰi/ | 扁担/piẽ tan/ | 个性/ko ɕin/ | 路费/ləu fei/ | 月桂/ye kuei/ |
| bedding | friendly | shoulder pole | personality | tolls | laurel |
| 妖怪/jau kuai/ | 灵泛/lin fan/ | 韭菜/tɕiəu tsʰai/ | 相片/ɕian pʰiẽ/ | 面片/miẽ pʰiẽ/ | 力气/li tɕʰi/ |
| monster | smart | leek | photo | noodle pieces | strength |
| 空气/kʰən tɕʰi/ | 洋气/jan tɕʰi/ | 眼睛/ŋan tɕin/ | 战士/tsɤ̃ sɿ/ | 忘记/wan tɕi/ | 学校/ɕio tɕiau/ |
| air | stylish | eye | warrior | forget | school |
| T1-T5 | T2-T5 | T3-T5 | T4-T5 | T5-T5 | T6-T5 |
| 刀豆/tau təu/ | 名字/min tsɿ/ | 姊妹/tsɿ mei/ | 菜豆/tsʰai təu/ | 夜饭/ja fan/ | 得罪/tə tsei/ |
| sword bean | name | sister | kidney bean | dinner | offend |
| 方便/fan piẽ/ | 油画/jəu fa/ | 马上/ma san/ | 笑话/ɕiau fa/ | 字号/tsɿ xau/ | 脚步/tɕio pu/ |
| convenience | paint | right now | joke | word size | footstep |
| 安静/ŋan tɕin/ | 桃树/tau ɕy/ | 老弟/lau ti/ | 气味/tɕʰi wei/ | 第二/ti ɤ/ | 北站/pə tsan/ |
| quiet | peach | younger brother | smell | second | north station |
| 东站/tən tsan/ | 毛病/mau pin/ | 小路/ɕiau ləu/ | 韵味/yn wei/ | 大事/ta sɿ/ | 约会/jo fei/ |
| east station | disease | footpath | taste | event | date |
| 鸡蛋/tɕi tan/ | 时候/sɿ xəu/ | 手艺/səu ȵi/ | 做梦/tsəu moŋ/ | 梦话/mən fa/ | 鸭蛋/ŋa tan/ |
| egg | time | skill | dreaming | sleep talk | duck egg |
| T1-T6 | T2-T6 | T3-T6 | T4-T6 | T5-T6 | T6-T6 |
| 清白/tsʰin pə/ | 黄历/faŋ li/ | 马脚/ma tɕio/ | 翼胛/ie ka/ | 是箇/sɿ ko/ | 蜡烛/la tsəu/ |
| cleanly | Chinese calendar | feet of horse | wing | repeatedly | candle |
| 衣服/i fu/ | 铅笔/yẽ pi/ | 等级/tən tɕi/ | 灶屋/tsau u/ | 事迹/sɿ tɕi/ | 泼辣/pʰo la/ |
| close | pencil | rank | kitchen | story | rude |
| 高级/kau tɕi/ | 麻雀/ma tɕʰio/ | 喜鹊/ɕi tɕʰio/ | 教室/tɕiau sɿ/ | 第一/ti i/ | 扎实/tsa sɿ/ |
| advanced | sparrow | magpie | classroom | first | robust |
| 亲戚/tɕʰin tɕʰi/ | 颜色/ŋan sɤ/ | 想法/ɕian fa/ | 宴席/iẽ ɕi/ | 痨药/lau jo/ | 指甲/tsɿ ka/ |
| relative | color | idea | banquet | poison | nail |
| 班级/pan tɕi/ | 蝴蝶/fu tʰie/ | 手续/səu səu/ | 性别/ɕin pʰie/ | 嫩肉/zən zəu/ | 白色/pɤ sɤ/ |
| class | butterfly | procedure | gender | tender meat | white |

**Table 1b** The disyllabic words of Chengdu dialect

| T1-T1 | T2-T1 | T3-T1 | T4-T1 |
| --- | --- | --- | --- |
| 东西 /toŋ ɕi/ | 蜜蜂 /mi foŋ/ | 点心 /tian ɕin/ | 亲家 /tɕʰin tɕia/ |
| item | bee | dim sum | relative |
| 乌鸦 /u ia/ | 菊花 /tɕy xua/ | 眼睛 /iæn tɕin/ | 舅妈 /tɕiəu ma/ |
| crow | chrysanthemum | eye | aunt |
| 啰嗦 /lo so/ | 南瓜 /læn kua/ | 手巾 /səu tɕin/ | 菜刀 /tsʰai tau/ |
| blurr | pumpkin | towel | kitchen knife |
| 香蕉 /ɕiaŋ tɕiau/ | 毛衣 /mau i/ | 哑巴 /ia pa/ | 衬衣 /tsʰən i/ |
| banana | sweater | dummy | shirts |
| 蜘蛛 /tsɿ tsu/ | 明天 /min tʰian/ | 紧张 /tɕin tsaŋ/ | 汽车 /tɕʰi tsʰe/ |
| spider | tomorrow | nervous | car |
| T1-T2 | T2-T2 | T3-T2 | T4-T2 |
| 冰雹 /pin pau/ | 裁缝 /tsʰai foŋ/ | 母牛 /mu ȵiəu/ | 臭虫 /tsʰəu tsʰoŋ/ |
| hail | tailor | cow | bug |
| 干粮 /kæn liaŋ/ | 蜡烛 /la tsu/ | 水泥 /suei ȵi/ | 算盘 /suæn pʰæn/ |
| food | candle | cement | abacus |
| 休息 /ɕiəu ɕi/ | 眉毛 /mei mau/ | 语言 /y ian/ | 自然 /zɿ zæn/ |
| rest | eyebrow | language | nature |
| 今年 /tɕin ȵian/ | 明年 /miŋ ȵian/ | 枕头 /tsən tʰəu/ | 凤凰 /foŋ xuaŋ/ |
| this year | next year | pillow | phoenix |
| 工人 /koŋ zən/ | 喉咙 /xəu loŋ/ | 主人 /tsu zən/ | 玉兰 /y læn/ |
| worker | throat | master | yulan |
| T1-T3 | T2-T3 | T3-T3 | T4-T3 |
| 肩膀 /tɕian paŋ/ | 柴火 /tsʰai xo/ | 水手 /suei səu/ | 道理 /tau li/ |
| shoulder | firewood | sailor | principle |
| 开水 /kʰai suei/ | 莲子 /lian tsɿ/ | 口水 /kʰəu suei/ | 右手 /iəu səu/ |
| boiled water | lotus seed | saliva | right hand |
| 蝌蚪 /kʰo təu/ | 朋友 /pʰoŋ iəu/ | 手掌 /səu tsaŋ/ | 战友 /tsæn iəu/ |
| tadpole | friend | palm | comrade |
| 清楚 /tɕʰin tsʰu/ | 读者 /tu tse/ | 左手 /tso səu/ | 对手 /tuei səu/ |
| clear | reader | left hand | opponent |
| 抄手 /tʰau səu/ | 芒果 /maŋ ko/ | 老鼠 /nau su/ | 户口 /fu kʰəu/ |
| wonton | mango | mouse | residence |
| T1-T4 | T2-T4 | T3-T4 | T4-T4 |
| 鸡蛋 /tɕi tæn/ | 肥皂 /fei tsau/ | 扁担 /pian tan/ | 照顾 /tsau ku/ |
| egg | soap | shoulder pole | take care |
| 家具 /tɕia tɕy/ | 学校 /ɕio ɕiau/ | 改善 /kai sæn/ | 个性 /ko ɕin/ |
| furniture | school | improve | personality |
| 师傅 /sɿ fu/ | 邮票 /iəu pʰiau/ | 讲究 /tɕiaŋ tɕiəu/ | 运气 /yn tɕʰi/ |
| teacher | stamp | attention | luck |
| 兄弟 /ɕioŋ ti/ | 萝卜 /lo pu/ | 韭菜 /tɕiəu tsʰai/ | 味道 /wei tau/ |
| brother | radishi | leek | taste |
| 妖怪 /iau kuai/ | 玫瑰 /mei kuei/ | 好处 /xau tsʰu/ | 故意 /ku i/ |
| monster | rose | advantage | on purpose |

**Table 1c** The disyllabic words of Fuzhou dialect

| T1-T1 | T1-T2 | T1-T3 | T1-T4 | T1-T5 |
| --- | --- | --- | --- | --- |
| 花生 /hua lɛiŋ/ | 窗帘 /tsʰouŋ nieŋ/ | 批纸 /pʰie ʒai/ | 铺盖 /pʰo kai/ | 兄弟 /hiaŋ nie/ |
| peanut | curtain | letter paper | bedding | brother |
| 家私 /ka li/ | 干粮 /kaŋ luoŋ/ | 鸡母 /kie mɔ/ | 生意 /sɛiŋ ei/ | 军事 /kuŋ nøy/ |
| furniture | food | hen | business | military |
| 乌龟 /u kui/ | 高粱 /kɔ luoŋ/ | 清楚 /tsʰiŋ ŋu/ | 消化 /siu xua/ | 冰雹 /piŋ pau/ |
| turtle | sorghum | clear | digest | hail |
| 西瓜 /sɛ ua/ | 衣裳 /i luɔŋ/ | 开水 /kʰa ʒui/ | 妖怪 /iu kuai/ | 鸡卵 /kie lɔuŋ/ |
| watermelon | clothes | boiled water | monster | egg |
| 先生 /siŋ ŋaŋ/ | 工人 /køŋ ŋiŋ/ | 猪母 /ty mɔ/ | 空气 /kʰuŋ kʰei/ | 师傅 /sa au/ |
| sir | worker | sow | air | teacher |
| T2-T1 | T2-T2 | T2-T3 | T2-T4 | T2-T5 |
| 桃花 /tʰɔ ua/ | 眉毛 /mi mɔ/ | 来往 /lai uoŋ/ | 明旦 /miŋ naŋ/ | 毛病 /mɔ βaŋ/ |
| peach blossom | eyebrow | contact | tomorrow | disease |
| 厨师 /to ly/ | 明年 /maŋ nieŋ/ | 莲子 /lɛiŋ ʒi/ | 奇怪 /ki kuai/ | 名誉 /miaŋ øy/ |
| chef | next year | lotus seed | strange | reputation |
| 头梳 /tʰau lœ/ | 便宜 /peiŋ ŋie/ | 芒果 /maŋ kɔ/ | 弹性 /taŋ ŋɛiŋ/ | 名字 /miaŋ ŋɛi/ |
| comb | cheap | mango | elasticity | name |
| 兰花 /laŋ ua/ | 糊涂 /hu tu/ | 茶水 /ta tsui/ | 详细 /suoŋ sa/ | 胰皂 /i ʒɔ/ |
| orchid | confused | tea | in detail | soap |
| 红蚣 /øyŋ ŋøyŋ/ | 葡萄 /pɔ lɔ/ | 牛母 /ŋu mɔ/ | 伶俐 /liŋ ŋɛi/ | 容易 /yŋ ŋɛi/ |
| centipede | grape | cow | smart | easy |
| T3-T1 | T3-T2 | T3-T3 | T3-T4 | T3-T5 |
| 苦瓜 /kʰu kua/ | 锁匙 /so lie/ | 洗水 /sɛ ʒuei/ | 姐妹 /tsia muoi/ | 好坏 /ho ŋai/ |
| bitter gourd | key | swim | sister | good and bad |
| 牡丹 /mu laŋ/ | 语言 /ŋy ŋyoŋ/ | 左手 /tso tsʰiu/ | 韭菜 /kiu ʒai/ | 买卖 /mɛ ma/ |
| peony | language | left hand | leek | buy and sell |
| 老鸦 /lɔ ua/ | 枕头 /tsieŋ nau/ | 草蜢 /tsʰau maŋ/ | 馆店 /kuaŋ naiŋ/ | 手艺 /tsʰiu niɛ/ |
| crow | pillow | grasshopper | restaurant | skill |
| 野鲜 /ia tsʰieŋ/ | 码头 /ma lau/ | 老鼠 /lo ʒy/ | 讨厌 /tʰɔ ieŋ/ | 本事 /poŋ nøy/ |
| fresh | pier | mouse | hate | ability |
| 火车 /hui ʒia/ | 主人 /tso nøyŋ/ | 海水 /hai ʒuoi/ | 海带 /hai ai/ | 好样 /hɔ yɔŋ/ |
| train | master | sea water | kelp | good sample |
| T4-T1 | T4-T2 | T4-T3 | T4-T4 | T4-T5 |
| 背心 /pui liŋ/ | 去年 /kʰɔ nieŋ/ | 露水 /lu ʒuei/ | 报告 /pɔ kɔ / | 破病 /pʰuai βaŋ/ |
| vest | last year | dew | report | fall ill |
| 菜刀 /tsʰai lɔ/ | 汽油 /kʰi iu / | 战友 /tsiɛŋ iu/ | 面布 /miŋ muɔ/ | 孝顺 /hau louŋ/ |
| kitchen knife | gasoline | comrade | towel | obedient |
| 秘书 /mi ʒy/ | 菜头 /tsʰai tau/ | 对手 /tɔy tsʰiu/ | 相片 /suɔŋ pʰieŋ/ | 战士 /tsiɛŋ ŋøy/ |
| secretary | turnip | opponent | photo | warrior |
| 桂花 /kiɛ ua/ | 劲头 /kiŋ tʰau/ | 燕鸟 /ieŋ nɛu/ | 世界 /sie kai/ | 看病 /kʰaŋ paŋ/ |
| osmanthus | strength | swallow | world | see a doctor |
| 丈夫 /touŋ muo/ | 算盘 /souŋ muaŋ/ | 报纸 /po ʒai/ | 退化 /tʰɔy hua/ | 印度 /in tou/ |
| husband | abacus | paper | degeneration | India |
| T5-T1 | T5-T2 | T5-T3 | T5-T4 | T5-T5 |
| 大方 /tuai kʰuɔŋ/ | 内行 /nøy ouŋ/ | 豆囝 /tau iaŋ/ | 大意 /tuai ɛi/ | 厉害 /li ʒai/ |
| generous | adept | soybean | general idea | sharp |
| 大衣 /tuai i/ | 状元 /tsɔŋ ŋuoŋ/ | 右手 /iu tsʰiu/ | 浪费 /loŋ hɛi/ | 第二 /tɛ nɛi/ |
| coat | scholar | right hand | waste | second |
| 是非 /sɿ hi/ | 自然 /tsy yoŋ/ | 户口 /hu ʒou/ | 受气 /siu kʰei/ | 命运 /miaŋ ŋouŋ/ |
| right and wrong | nature | residence | be bullied | destiny |
| 外家 /ŋie a/ | 丈侬 /tuɔŋ nøyŋ/ | 电影 /tieŋ ŋiŋ/ | 忘记 /mɛ kei/ | 电话 /tieŋ uɑ/ |
| mother home | father-in-law | movie | forget | phone |
| 杜鹃 /tu kyoŋ/ | 外行 /ŋui ouŋ/ | 老虎 /lau u/ | 运气 /oŋ kʰɛi/ | 自动 /tsy louŋ/ |
| cuckoo | layman | tiger | luck | automatic |

**Table 1d** The disyllabic words of Xiamen dialect

| T1-T1 | T1-T2 | T1-T3 | T1-T4 | T1-T5 |
| --- | --- | --- | --- | --- |
| 乌鸦 /ɔ ku/ | 高粱 /ko liaŋ/ | 骹趾 /kʰa tsai/ | 甘蔗 /kam tsia/ | 安静 /an tsɪŋ / |
| crow | sorghum | toe | sugarcane | quiet |
| 先生 /siɛŋ sĩ/ | 棺材 /kuã tsʰa/ | 猪母 /ti bu/ | 天气 /tʰĩ kʰi/ | 鸡宿 /kue siu/ |
| sir | coffin | sow | weather | henhouse |
| 交通 /kau tʰɔŋ/ | 今年 /kin nĩ/ | 批纸 /pʰue tsua/ | 相信 /siɔŋ sin/ | 铺盖 /pʰɔ tsʰɔ/ |
| traffic | this year | letter paper | trust | bedding |
| 猪公 /ti ko/ | 工人 /kaŋ laŋ/ | 鸡母 /kue bu/ | 消化 /siau hua / | 兄弟 /hĩa ti/ |
| boar | worker | hen | digest | brother |
| 蜘蛛 /ti tu/ | 金钱 /kin tsĩ/ | 清楚 /tsʰiŋ tsʰo/ | 妖怪 /iau kuai / | 方便 /hɔŋ piɛn/ |
| spider | money | clear | monster | convenient |
| T2-T1 | T2-T2 | T2-T3 | T2-T4 | T2-T5 |
| 楼梯 /lau tʰui/ | 裁缝 /tsʰai hɔŋ/ | 茶水 /te tsui/ | 奇怪 /ki kuai/ | 毛病 /mɔ̃ pʰĩ/ |
| ladder | tailor | tea | strange | disease |
| 桃花 /tʰo hue/ | 明年 /mẽ nĩ/ | 柴火 /tsʰa he/ | 详细 /siɔŋ se/ | 门路 /mŋ lɔ/ |
| peach blossom | next year | firewood | detail | social connection |
| 行家 /haŋ ka/ | 葫芦 /hɔ lɔ/ | 扶手 /hu tsʰiu/ | 伶俐 /lɪŋ li/ | 名誉 /mĩa u/ |
| expert | gourd | handrail | clever | reputation |
| 盐巴 /iam pa/ | 葡萄 /pʰu tʰau/ | 牛母 /ɡu bu/ | 男性 /lam sɪŋ/ | 时候 /si tsun/ |
| salt | grape | cow | male | time |
| 茶瓯 /te au/ | 锄头 /ti tʰau/ | 泅水 /siu tsui/ | 难处 /nan tsʰu/ | 容易 /iɔŋ i/ |
| cup | hoe | swim | difficulty | easy |
| T3-T1 | T3-T2 | T3-T3 | T3-T4 | T3-T5 |
| 点心 /tiam sim/ | 锁匙 /so si/ | 滚水 /kun tsui/ | 讲究 /kɔŋ kiu/ | 马上 /ma siɔŋ/ |
| dim sim | key | boiled water | pay attention to | right away |
| 牡丹 /bɔ tan/ | 语言 /ɡu ɡiɛn/ | 手掌 /tsʰiu tsim/ | 否势 /pʰai se/ | 改善 /kai siɛn/ |
| peony | language | palm | shy | improve |
| 火车 /hue tsʰia/ | 马荠 /be tsi/ | 手指 /tsʰiu tsai/ | 韭菜 /ku tsʰai/ | 姐妹 /tsi be/ |
| train | water chestnut | finger | leek | sister |
| 水仙 /tsui siɛn/ | 海棠 /hai tɔŋ/ | 哑口 /e kau/ | 囝婿 /kĩa sai/ | 买卖 /bue bue/ |
| narcissus | begonia | dummy | son-in-law | bargain |
| 紧张 /kin tĩu/ | 水泥 /tsui nĩ/ | 老虎 /lau hɔ/ | 好处 /ho tsʰu/ | 手艺 /tsʰiu ɡe/ |
| nervous | cement | tiger | advantage | skill |
| T4-T1 | T4-T2 | T4-T3 | T4-T4 | T4-T5 |
| 菜刀 /tsʰai to/ | 报酬 /po siu/ | 厝顶 /tsʰu tiŋ/ | 放屁 /paŋ pʰui/ | 故事 /kɔ su/ |
| kitchen knife | payment | roof | fart | story |
| 厝边 /tsʰu pĩ/ | 臭虫 /tsʰau tʰaŋ/ | 嘴齿 /tsʰui kʰi/ | 相片 /siɔŋ pʰĩ/ | 气味 /kʰi bi/ |
| neighbor | bug | tooth | photo | smell |
| 秘书 /pi su/ | 算盘 /sŋ pũa/ | 正手 /tsĩa tsʰiu/ | 看看 /kʰua kʰua/ | 战士 /tsiɛn su/ |
| secretary | abacus | right hand | looking | warrior |
| 桂花 /kui hue/ | 菜头 /tsʰai tʰau/ | 战友 /tsiɛn iu/ | 性地 /sɪŋ te/ | 做梦 /tsue baŋ/ |
| osmanthus | turnip | comrade | temper | dreaming |
| 汽车 /kʰi tsʰia/ | 跳槽 /tʰiau tso/ | 倒手 /to tsʰiu/ | 告示 /ko si/ | 看病 / kʰuã pĩ/ |
| car | job-hopping | left hand | bulletin | see a doctor |
| T5-T1 | T5-T2 | T5-T3 | T5-T4 | T5-T5 |
| 背心 /pe sim/ | 状元 /tsɔŋ ɡuan/ | 父母 /pe bu/ | 味素 /bi sɔ/ | 动静 /tɔŋ tsɪŋ/ |
| vest | scholar | parents | smell | movement |
| 大方 /tua hɔŋ/ | 凤凰 /hɔŋ hɔŋ/ | 露水 /lɔ tsui/ | 外气 /ɡua kʰi/ | 豆腐 /tau hu/ |
| generous | phoenix | dew | stylish | tofu |
| 外家 /ɡua ke/ | 虭螂 /tɔ kau/ | 户口 /hɔ kau/ | 运气 /un kʰi/ | 厉害 /li hai/ |
| mother home | mantis | residence | luck | sharp |
| 电珠 /tiɛn tsu/ | 丈人 /tĩu laŋ/ | 电影 /tiɛn iã/ | 面布 /bin pɔ/ | 自动 /tsu tɔŋ/ |
| bulb | father-in-law | movie | towel | automatic |
| 外衫 /ɡua sã/ | 自由 /tsu iu/ | 斗蚓 /tɔ un/ | 饭店 /pŋ tiam/ | 第二 /te li/ |
| coat | freedom | earthworm | restaurant | second |

## Supplementary Table 2

**Table 2a** Tukey adjusted post-hoc test of the pitch slope (linear trend) of T1 in σ(2) in Changsha dialect between tonal contexts.

| Pitch parameter | Tonal combination | β | *SE* | *df* | *t* | *p* |
| --- | --- | --- | --- | --- | --- | --- |
| Slope | T1-T1 to T2-T1 | 0.03 | 0.13 | 49 | 0.25 | 1.000 |
|  | T1-T1 to T3-T1 | 0.04 | 0.13 | 49 | 0.28 | 1.000 |
|  | T1-T1 to T4-T1 | 0.22 | 0.13 | 49 | 1.64 | 0.578 |
|  | T1-T1 to T5-T1 | -0.17 | 0.13 | 49 | -1.29 | 0.789 |
|  | T1-T1 to T6-T1 | 0.21 | 0.13 | 49 | 1.58 | 0.617 |
|  | T2-T1 to T3-T1 | 0.00 | 0.13 | 49 | 0.03 | 1.000 |
|  | T2-T1 to T4-T1 | 0.19 | 0.13 | 49 | 1.39 | 0.733 |
|  | T2-T1 to T5-T1 | -0.21 | 0.13 | 49 | -1.54 | 0.641 |
|  | T2-T1 to T6-T1 | 0.18 | 0.13 | 49 | 1.33 | 0.769 |
|  | T3-T1 to T4-T1 | 0.18 | 0.13 | 49 | 1.36 | 0.748 |
|  | T3-T1 to T5-T1 | -0.21 | 0.13 | 49 | -1.57 | 0.625 |
|  | T3-T1 to T6-T1 | 0.17 | 0.13 | 49 | 1.30 | 0.783 |
|  | T4-T1 to T5-T1 | -0.39 | 0.13 | 49 | -2.93 | 0.055 |
|  | T4-T1 to T6-T1 | -0.01 | 0.13 | 49 | -0.06 | 1.000 |
|  | T5-T1 to T6-T1 | 0.38 | 0.13 | 49 | 2.87 | 0.063 |

**Table 2b** Tukey adjusted post-hoc test of the pitch slope (linear trend) of T4 in σ(2) in Changsha dialect between tonal contexts. Numbers in boldface indicate significant findings.

| Pitch parameter | Tonal combination | β | *SE* | *df* | *t* |  | *p* |
| --- | --- | --- | --- | --- | --- | --- | --- |
| Slope | T1-T4 to T2-T4 | -0.08 | 0.15 | 34 | -0.56 |  | 0.993 |
|  | T1-T4 to T3-T4 | 0.13 | 0.15 | 34 | 0.85 |  | 0.955 |
|  | T1-T4 to T4-T4 | 0.39 | 0.15 | 34 | 2.61 |  | 0.123 |
|  | **T1-T4 to T5-T4** | **-0.48** | **0.15** | **34** | **-3.18** |  | **<.05 *** |
|  | T1-T4 to T6-T4 | 0.11 | 0.15 | 34 | 0.72 |  | 0.978 |
|  | T2-T4 to T3-T4 | 0.21 | 0.15 | 34 | 1.41 |  | 0.720 |
|  | **T2-T4 to T4-T4** | **0.48** | **0.15** | **34** | **3.17** |  | **<.05 *** |
|  | T2-T4 to T5-T4 | -0.40 | 0.15 | 34 | -2.63 |  | 0.119 |
|  | T2-T4 to T6-T4 | 0.19 | 0.15 | 34 | 1.28 |  | 0.795 |
|  | T3-T4 to T4-T4 | 0.27 | 0.15 | 34 | 1.76 |  | 0.506 |
|  | **T3-T4 to T5-T4** | **-0.61** | **0.15** | **34** | **-4.04** |  | **< .01 **** |
|  | T3-T4 to T6-T4 | -0.02 | 0.15 | 34 | -0.13 |  | 1.000 |
|  | **T4-T4 to T5-T4** | **-0.88** | **0.15** | **34** | **-5.79** |  | **<.001 ***** |
|  | T4-T4 to T6-T4 | -0.29 | 0.15 | 34 | -1.89 |  | 0.425 |
|  | **T5-T4 to T6-T4** | **0.59** | **0.15** | **34** | **3.90** |  | **< .01 **** |

*Note*. Boldface indicates significant findings. * *p* < .05. ** *p* < .01. *** *p* < .001

**Table 2c** Tukey adjusted post-hoc test of the pitch slope (linear trend) of T6 in σ(2) in Changsha dialect between tonal contexts. Numbers in boldface indicate significant findings.

| Pitch parameter | Tonal combination | β | *SE* | *df* | *t* |  | *p* |
| --- | --- | --- | --- | --- | --- | --- | --- |
| Slope | T1-T6 to T2-T6 | -0.06 | 0.11 | 35 | -0.57 |  | 0.993 |
|  | T1-T6 to T3-T6 | 0.04 | 0.11 | 35 | 0.32 |  | 1.000 |
|  | T1-T6 to T4-T6 | 0.03 | 0.11 | 35 | 0.25 |  | 1.000 |
|  | **T1-T6 to T5-T6** | **-0.57** | **0.11** | **35** | **-5.10** |  | **< .001 ***** |
|  | T1-T6 to T6-T6 | 0.28 | 0.11 | 35 | 2.46 |  | 0.165 |
|  | T2-T6 to T3-T6 | 0.10 | 0.11 | 35 | 0.89 |  | 0.947 |
|  | T2-T6 to T4-T6 | 0.09 | 0.11 | 35 | 0.82 |  | 0.963 |
|  | **T2-T6 to T5-T6** | **-0.51** | **0.11** | **35** | **-4.53** |  | **< .01 **** |
|  | **T2-T6 to T6-T6** | **0.34** | **0.11** | **35** | **3.03** |  | **< .05 *** |
|  | T3-T6 to T4-T6 | -0.01 | 0.11 | 35 | -0.07 |  | 1.000 |
|  | **T3-T6 to T5-T6** | **-0.61** | **0.11** | **35** | **-5.42** |  | **< .001 ***** |
|  | T3-T6 to T6-T6 | 0.24 | 0.11 | 35 | 2.14 |  | 0.292 |
|  | **T4-T6 to T5-T6** | **-0.60** | **0.11** | **35** | **-5.35** |  | **< .001 ***** |
|  | T4-T6 to T6-T6 | 0.25 | 0.11 | 35 | 2.21 |  | 0.259 |
|  | **T5-T6 to T6-T6** | **0.85** | **0.11** | **35** | **7.56** |  | **< .001 ***** |

*Note*. Boldface indicates significant findings. * *p* < .05. ** *p* < .01. *** *p* < .001

## Supplementary Table 3

**Table 3a** Tukey adjusted post-hoc test of the pitch slope (linear trend) of T2 in σ(2) in Chengdu dialect between tonal contexts. Numbers in boldface indicate significant findings.

| Pitch parameter | Tonal combination | β | *SE* | *df* | *t* |  | *p* |
| --- | --- | --- | --- | --- | --- | --- | --- |
| Slope | **T1-T2 to T2-T2** | **-2.85** | **0.28** | **26** | **-10.33** |  | **< .001 ***** |
|  | T1-T2 to T3-T2 | 0.31 | 0.28 | 26 | 1.11 |  | 0.690 |
|  | T1-T2 to T4-T2 | -0.63 | 0.28 | 26 | -2.28 |  | 0.129 |
|  | **T2-T2 to T3-T2** | **3.15** | **0.28** | **26** | **11.44** |  | **< .001 ***** |
|  | **T2-T2 to T4-T2** | **2.22** | **0.28** | **26** | **8.05** |  | **< .001 ***** |
|  | **T3-T2 to T4-T2** | **-0.93** | **0.28** | **26** | **-3.39** |  | **< .05 *** |

*Note*. Boldface indicates significant findings. * *p* < .05. *** *p* < .001

**Table 3b** Tukey adjusted post-hoc test of the pitch slope (linear trend) of T3 in σ(2) in Chengdu dialect between tonal contexts. Numbers in boldface indicate significant findings.

| Pitch parameter | Tonal combination | β | *SE* | *df* | *t* |  | *p* |
| --- | --- | --- | --- | --- | --- | --- | --- |
| Slope | **T1-T3 to T2-T3** | **-0.72** | **0.26** | **33** | **-2.75** |  | **< .05 *** |
|  | T1-T3 to T3-T3 | 0.08 | 0.26 | 33 | 0.30 |  | 0.990 |
|  | T1-T3 to T4-T3 | -0.64 | 0.26 | 33 | -2.45 |  | 0.088 |
|  | **T2-T3 to T3-T3** | **0.80** | **0.26** | **33** | **3.05** |  | **< .05 *** |
|  | T2-T3 to T4-T3 | 0.08 | 0.26 | 33 | 0.30 |  | 0.990 |
|  | **T3-T3 to T4-T3** | **-0.72** | **0.26** | **33** | **-2.75** |  | **< .05*** |

*Note.* Boldface indicates significant findings. * *p* < .05.

**Table 3c** Tukey adjusted post-hoc test of the pitch slope (linear trend) of T4 in σ(2) in Chengdu dialect between tonal contexts.

| Pitch parameter | Tonal combination | β | *SE* | *df* | *t* | *p* |
| --- | --- | --- | --- | --- | --- | --- |
| Slope | T1-T3 to T2-T3 | -0.38 | 0.41 | 27 | -0.94 | 0.783 |
|  | T1-T3 to T3-T3 | 0.39 | 0.41 | 27 | 0.96 | 0.774 |
|  | T1-T3 to T4-T3 | -0.68 | 0.41 | 27 | -1.68 | 0.351 |
|  | T2-T3 to T3-T3 | 0.77 | 0.41 | 27 | 1.90 | 0.253 |
|  | T2-T3 to T4-T3 | -0.30 | 0.41 | 27 | -0.74 | 0.879 |
|  | T3-T3 to T4-T3 | -1.07 | 0.41 | 27 | -2.64 | 0.061 |

## Supplementary Table 4

**Table 4a** Tukey adjusted post-hoc test of the pitch slope (linear trend) of T1 in σ(1) in Fuzhou dialect between tonal contexts. Numbers in boldface indicate significant findings.

| Pitch parameter | Tonal combination | β | *SE* | *df* | *t* |  | *p* |
| --- | --- | --- | --- | --- | --- | --- | --- |
| Slope | T1-T1 to T1-T2 | -0.40 | 0.47 | 45 | -0.85 |  | 0.914 |
|  | T1-T1 to T1-T3 | 1.27 | 0.47 | 45 | 2.70 |  | 0.071 |
|  | **T1-T1 to T1-T4** | **2.16** | **0.47** | **45** | **4.61** |  | **< .001 ***** |
|  | **T1-T1 to T1-T5** | **2.45** | **0.47** | **45** | **5.21** |  | **< .001 ***** |
|  | **T1-T2 to T1-T3** | **1.66** | **0.47** | **45** | **3.54** |  | **< .01 **** |
|  | **T1-T2 to T1-T4** | **2.56** | **0.47** | **45** | **5.45** |  | **< .001 ***** |
|  | **T1-T2 to T1-T5** | **2.84** | **0.47** | **45** | **6.06** |  | **< .001 ***** |
|  | T1-T3 to T1-T4 | 0.90 | 0.47 | 45 | 1.91 |  | 0.326 |
|  | T1-T3 to T1-T5 | 1.18 | 0.47 | 45 | 2.51 |  | 0.105 |
|  | T1-T4 to T1-T5 | 0.28 | 0.47 | 45 | 0.60 |  | 0.974 |

*Note.* Boldface indicates significant findings. ** *p* < .01. *** *p* < .001

**Table 4b** Tukey adjusted post-hoc test of the pitch slope (linear trend) of T4 in σ(1) in Fuzhou dialect between tonal contexts. Numbers in boldface indicate significant findings.

| Pitch parameter | Tonal combination | β | *SE* | *df* | *t* |  | *p* |
| --- | --- | --- | --- | --- | --- | --- | --- |
| Slope | T4-T1 to T4-T2 | -0.17 | 0.30 | 33 | -0.57 |  | 0.978 |
|  | **T4-T1 to T4-T3** | **1.63** | **0.30** | **33** | **5.52** |  | **< .001 ***** |
|  | **T4-T1 to T4-T4** | **2.24** | **0.30** | **33** | **7.59** |  | **< .001 ***** |
|  | **T4-T1 to T4-T5** | **2.59** | **0.30** | **33** | **8.77** |  | **< .001 ***** |
|  | **T4-T2 to T4-T3** | **1.80** | **0.30** | **33** | **6.09** |  | **< .001 ***** |
|  | **T4-T2 to T4-T4** | **2.41** | **0.30** | **33** | **8.16** |  | **< .001 ***** |
|  | **T4-T2 to T4-T5** | **2.76** | **0.30** | **33** | **9.34** |  | **< .001 ***** |
|  | T4-T3 to T4-T4 | 0.61 | 0.30 | 33 | 2.07 |  | 0.256 |
|  | **T4-T3 to T4-T5** | **0.96** | **0.30** | **33** | **3.26** |  | **< .05 *** |
|  | T4-T4 to T4-T5 | 0.35 | 0.30 | 33 | 1.18 |  | 0.761 |

*Note*. Boldface indicates significant findings. * *p* < .05. *** *p* < .001.

**Table 4c** Tukey adjusted post-hoc test of the pitch slope (linear trend) of T5 in σ(1) in Fuzhou dialect between tonal contexts. Numbers in boldface indicate significant findings.

| Pitch parameter | Tonal combination | β | *SE* | *df* | *t* |  | *p* |
| --- | --- | --- | --- | --- | --- | --- | --- |
| Slope | T5-T1 to T5-T2 | -0.36 | 0.42 | 48 | -0.87 |  | 0.710 |
|  | T5-T1 to T5-T3 | 0.85 | 0.42 | 48 | 2.02 |  | 0.068 |
|  | **T5-T1 to T5-T4** | **1.26** | **0.42** | **48** | **3.00** |  | **< .05 *** |
|  | **T5-T1 to T5-T5** | **1.67** | **0.42** | **48** | **4.00** |  | **< .01 **** |
|  | **T5-T2 to T5-T3** | **1.21** | **0.42** | **48** | **2.89** |  | **< .05 *** |
|  | **T5-T2 to T5-T4** | **1.62** | **0.42** | **48** | **3.87** |  | **< .01 **** |
|  | **T5-T2 to T5-T5** | **2.04** | **0.42** | **48** | **4.87** |  | **< .001 ***** |
|  | T5-T3 to T5-T4 | 0.41 | 0.42 | 48 | 0.98 |  | 0.986 |
|  | T5-T3 to T5-T5 | 0.83 | 0.42 | 48 | 1.98 |  | 0.192 |
|  | T5-T4 to T5-T5 | 0.42 | 0.42 | 48 | 1.00 |  | 0.069 |

*Note*. Boldface indicates significant findings. * *p* < .05. ** *p* < .01. *** *p* < .001

**Table 4d** Tukey adjusted post-hoc test of the pitch height (intercept) of T2 in σ(w) in Fuzhou dialect between tonal contexts. Numbers in boldface indicate significant findings.

| Pitch parameter | Tonal combination | β | *SE* | *df* | *t* |  | *p* |
| --- | --- | --- | --- | --- | --- | --- | --- |
| Intercept | T2-T1 to T2-T2 | 0.61 | 0.17 | 40 | 3.487 |  | 0.401 |
|  | **T2-T1 to T2-T3** | **1.00** | **0.17** | **40** | **5.75** |  | **< .001 ***** |
|  | **T2-T1 to T2-T4** | **1.63** | **0.17** | **40** | **9.33** |  | **< .001 ***** |
|  | **T2-T1 to T2-T5** | **1.76** | **0.17** | **40** | **10.07** |  | **< .001 ***** |
|  | T2-T2 to T2-T3 | 0.40 | 0.17 | 40 | 2.26 |  | 0.178 |
|  | **T2-T2 to T2-T4** | **1.02** | **0.17** | **40** | **5.84** |  | **< .001 ***** |
|  | **T2-T2 to T2-T5** | **1.15** | **0.17** | **40** | **6.59** |  | **< .001 ***** |
|  | **T2-T3 to T2-T4** | **0.62** | **0.17** | **40** | **3.58** |  | **< .01 **** |
|  | **T2-T3 to T2-T5** | **0.75** | **0.17** | **40** | **4.32** |  | **< .01 **** |
|  | T2-T4 to T2-T5 | 0.13 | 0.17 | 40 | 0.75 |  | 0.945 |

*Note*. Boldface indicates significant findings. ** *p* < .01. *** *p* < .001.
